# Characterization of an acid rock drainage microbiome and transcriptome at the Ely Copper Mine Superfund site

**DOI:** 10.1371/journal.pone.0237599

**Published:** 2020-08-12

**Authors:** Lesley-Ann Giddings, George Chlipala, Kevin Kunstman, Stefan Green, Katherine Morillo, Kieran Bhave, Holly Peterson, Heather Driscoll, Mark Maienschein-Cline

**Affiliations:** 1 Department of Chemistry & Biochemistry, Middlebury College, Middlebury, Vermont, United States of America; 2 Department of Chemistry, Smith College, Northampton, Massachusetts, United States of America; 3 Research Resources Center, University of Illinois at Chicago, Chicago, Illinois, United States of America; 4 Department of Geology, Guilford College, Greensboro, North Carolina, United States of America; 5 Vermont Genetics Network, Department of Biology, Norwich University, Northfield, Vermont, United States of America; University of Utah, UNITED STATES

## Abstract

The microbial oxidation of metal sulfides plays a major role in the formation of acid rock drainage (ARD). We aimed to broadly characterize the ARD at Ely Brook, which drains the Ely Copper Mine Superfund site in Vermont, USA, using metagenomics and metatranscriptomics to assess the metabolic potential and seasonal ecological roles of microorganisms in water and sediment. Using Centrifuge against the NCBI “nt” database, ~25% of reads in sediment and water samples were classified as acid-tolerant Proteobacteria (61 ± 4%) belonging to the genera *Pseudomonas* (2.6–3.3%), *Bradyrhizobium* (1.7–4.1%), and *Streptomyces* (2.9–5.0%). Numerous genes (12%) were differentially expressed between seasons and played significant roles in iron, sulfur, carbon, and nitrogen cycling. The most abundant RNA transcript encoded the multidrug resistance protein Stp, and most expressed KEGG-annotated transcripts were involved in amino acid metabolism. Biosynthetic gene clusters involved in secondary metabolism (BGCs, 449) as well as metal- (133) and antibiotic-resistance (8501) genes were identified across the entire dataset. Several antibiotic and metal resistance genes were colocalized and coexpressed with putative BGCs, providing insight into the protective roles of the molecules BGCs produce. Our study shows that ecological stimuli, such as metal concentrations and seasonal variations, can drive ARD taxa to produce novel bioactive metabolites.

## Introduction

**Fig 1:**
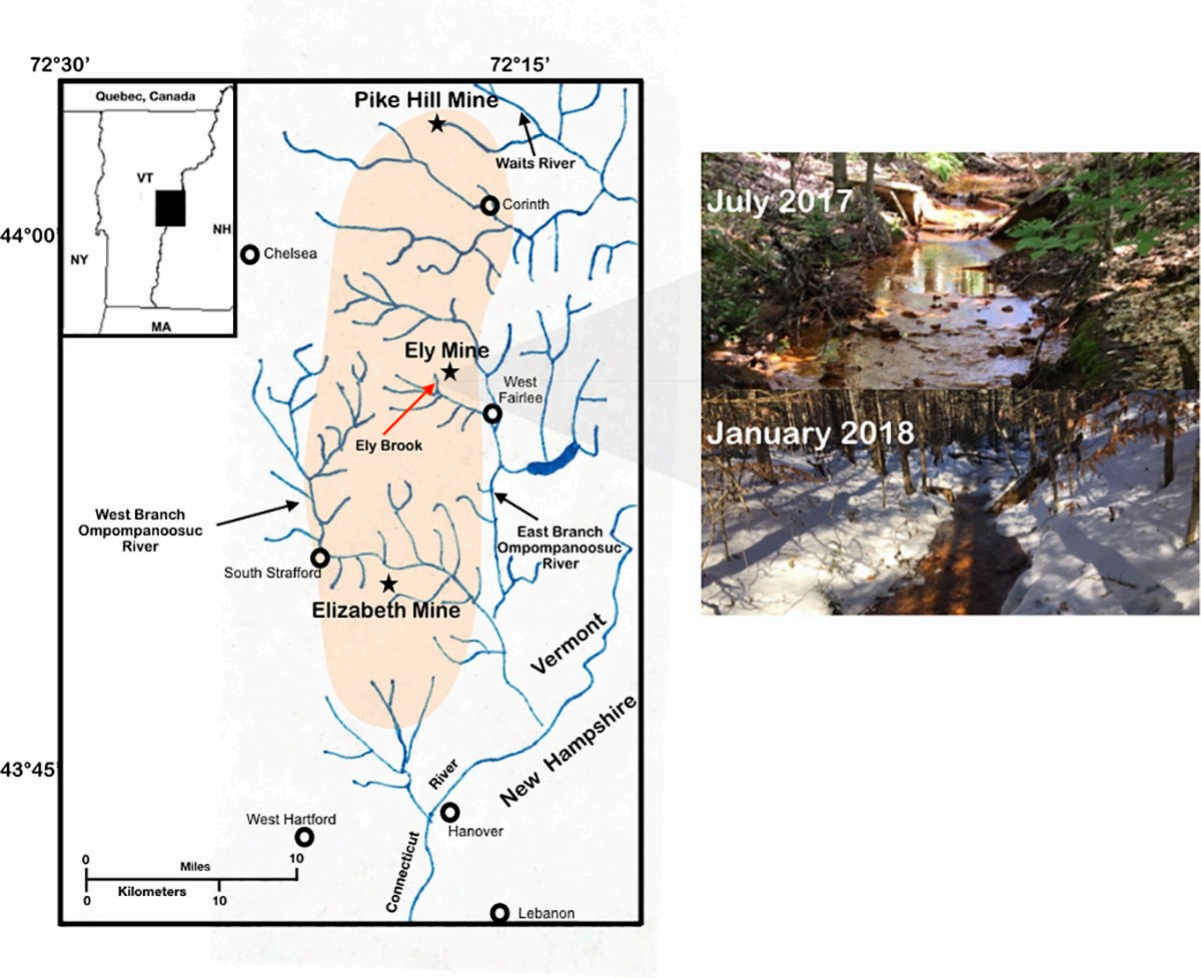
Vermont copper belt. Map of Elizabeth Mine, Ely Copper Mine, and Pike Hill Copper Mine (represented by stars) [4]. Circles represent nearby towns and the Ely Brook (EB-90M) study site is indicated by a red arrow. The pictures on the right show the EB-90M study site on July 28^th^, 2017 and January 14^th^, 2018.

During the 19^th^ and 20^th^ centuries, the mining industry exploited Vermont’s copper belt in Orange County (Fig 1), after which several copper mines were abandoned and left to accumulate acid rock drainage (ARD) [1]. ARD is the outflow of acidic water from mining regions containing metal-sulfide-rich rocks. When metal sulfides are exposed to water and oxygen, hydronium and sulfate ions are produced, lowering the pH of the water. Toxic levels of Cu, Fe, Zn, and Pb, leaching from pyrrhotite-rich, Besshi-type sulfide deposits [2] have adversely affected the water quality and aquatic biodiversity in the copper belt [3]. This process is further accelerated by the presence of acidophilic, sulfur and/or iron-oxidizing bacteria, which quickly convert insoluble sulfides to soluble sulfate ions and Fe^2+^ to Fe^3+^, the predominant, soluble form of iron at acidic pH. Due to metals and acidic waters contaminating local streams, mines in this region have been placed on the Superfund National Priorities List by the Environmental Protection Agency (EPA).

Microorganisms in metal-contaminated environments evolve unique genes conferring resistance to heavy metals [5, 6] and/or antibiotics [7–9] to maintain cellular homeostasis. Metal resistance genes (MRGs), some of which are antibiotic-resistant based on having similar mechanisms of action [8], can induce the biosynthesis of secondary metabolites to scavenge metals. For example, *Cupriavidus metallidurans*, originally isolated from industrial sludge [10], is both heavy metal- and antibiotic-resistant and expresses biosynthetic gene clusters (BGCs) involved in the production of a variety of secondary metabolites, including Fe^3+^-binding staphyloferrin B [11, 12]. Several bioactive microbial natural products have been isolated from mining environments [13], such as the berkeleylactones, potent fungal antibiotics isolated from the copper-rich Berkeley Pit in Butte, MT [14]. MRGs and antibiotic resistance genes have also been identified within biosynthetic gene clusters (BGCs) dedicated to secondary metabolism [15]. Thus, the coclustering of these resistance genes can be used to bioprospect metal-polluted environments for novel secondary metabolites and understand the stressors that trigger their production, providing insight into their bioactivity.

To assess the potential of ARD to produce bioactive secondary metabolites, we characterized the water and sediment associated with ARD at the Superfund site Ely Copper Mine. Both water and sediment at this mine have been affected by ARD (pH > 3) and high metal concentrations (e.g., up to 1,560 μg/L Cu) [3]. In 2010, dissolved Cu concentrations in the water and sediment at Ely Copper Mine exceeded the aquatic health criteria by 45–222 and 7–40 times, respectively [3]. Thus, we sampled water and sediment at the Ely Brook, a confluence of clean water and upstream tributaries that drain the mine [3], and used shotgun metagenomics and metatranscriptomics to characterize the ARD microbiome, including community structure and diversity, and the genes involved in secondary metabolism as well as heavy metal and antibiotic resistance.

Acid rock drainage sites rich in copper tailings are generally inhabited by acidophilic iron- and sulfur-oxidizing microorganisms, such as species of *Leptospirillum*, *Acidithiobacillus*, *Acidiphilium*, and *Thiobacillus* [16–18]. Based on the high acidity and metal concentrations in Ely Brook [3], we hypothesized that similar species would dominate in Ely Brook. In this study, we aimed to 1) describe the acidophilic, iron- and sulfur-oxidizing chemolithoautotrophs and heterotrophs that likely dominate the water and sediment at Ely Brook and 2) link the microbiome to actively expressed genes, especially those involved in metal transport and the production of bioactive secondary metabolites in this metal-rich extreme environment. Samples were collected in summer and winter to identify seasonal differences that inform community dynamics as well as how environmental stimuli affect gene expression. This work represents the first metagenomic and metatranscriptomic study of an acid rock microbiome within the Vermont copper belt.

## Materials and methods

### Study site and sample collection

On July 28^th^, 2017 and January 14^th^, 2018, Ely Brook (43°55’9” N, 72°17’11” W), 90 m upstream from the mouth of the brook (EB-90M), was sampled along with unsaturated sediment (10 cm deep) from the bank. Five water samples at EB-90M were filtered through Sterivex-GP 0.22-μm polyethanesulfone filters (MilliporeSigma; St. Louis, MO) using a varistaltic pump to collect DNA for sequencing and frozen on dry ice. Additional water samples were collected in HPDE Packers (Thermofisher; Waltham, MA) and a subset were filtered and/or preserved with either sulfuric acid (for organic carbon analyses) or nitric acid (for elemental analyses). Three sediment samples were also collected in sterile containers and frozen on dry ice for RNA and DNA extraction. Sediment was also collected for elemental analyses, and the physicochemical properties of samples were analyzed on-site or in a laboratory. A total of 16 samples were collected in July and January. See the supporting information (S1 Data).

### DNA & RNA extraction, library construction, metagenomic and metatranscriptomic sequencing

DNA was extracted from water (≥1 L) and sediment samples (0.25 g) using the DNeasy PowerWater Sterivex^®^ and PowerSoil^®^ DNA Isolation kits (Mo Bio Laboratories, Inc.; Carlsbad, CA) according to the manufacturer’s instructions. RNA was extracted from sediment (2.0 g) using the RNeasy PowerSoil^®^ Total RNA Isolation kit (Mo Bio Laboratories, Inc.; Carlsbad, CA). Library preparation and sequencing were performed at the University of Illinois at Chicago Sequencing Core (UICSQC). Details on nucleotide extraction and library preparation, sequencing, and quality assessment can be found in the Supporting Information. DNA and RNA library sequencing were performed on Illumina NextSeq500 and NextSeq500 high-output kits, respectively, with paired-end 150 base sequencing reads.

### Taxonomic annotation and quantification

Reads were mapped to the NCBI nucleotide database (nt) via Centrifuge [19] using nucleotide BLAST (v2.2.29+), retaining alignments of at least 500 bp and an E-value < 10^−4^ [20, 21]. Taxonomic annotations per DNA fragment (read pair) were obtained and summarized across all read pairs to create counts per taxon. MEGAN’s Least Common Ancestor (LCA) algorithm (i.e., blast2lca tool) [22] was used to determine the taxonomic annotation for each read pair and taxonomic summaries were generated from the superkingdom to species levels. Raw counts were normalized to fractional counts for relative abundance.

### Differential analysis of microbial taxonomic summaries

To assess seasonal microbial differences, taxonomic summaries were split into the following superkingdoms (sk) and kingdom (k), respectively: sk_Bacteria, sk_Archaea, and k_Fungi. Rare or sparse taxa with <1000 total sequence counts across all samples and ten counts in at least three samples were filtered from each “kingdom-specific” table. Data were normalized to the total sequence counts prior to filtering, and differential analyses on each taxonomic summary were performed separately in edgeR [23]. Differential statistics (log_2_fold-changes and p-values) were computed for each taxon comparing season and sample type separately using the raw counts from the taxonomic annotation and quantification. In all cases, p-values were adjusted for multiple testing using the false discovery rate (FDR) correction (q-values) of Benjamini and Hochberg [24].

### Metagenomic assembly and annotation of open reading frames

Metagenomic assembly was performed using the Spades assembler v3.11.1 [25] on raw Illumina reads from all DNA samples with the multiple metagenomics “—meta” option specified. Default parameters were used unless specified. Coverage levels were assessed by mapping raw Illumina reads to the contigs with BWA-MEM v0.7.15 [26] using default parameters and computing the coverage as the number of reads aligning per contig times the length of each read divided by the length of the contig. Contigs were filtered to have a minimum length of 1000 bp, containing non-repetitive sequences (2-base Shannon entropy > 0.85) as well as greater than zero (0) coverage from an independent alignment of raw reads. Putative taxonomic annotation was performed using a local blastn search, v2.2.29+, with default parameters against NCBI “nt”, retaining alignments of at least 500 bp and an E-value < 10^−4^ [20, 21]. BLAST analyses were then summarized using MEGAN’s blast2lca tool [22] using default parameters.

Prokka [27] was used to detect and annotate functional genes/open reading frames (ORFs) present in contigs using the parameter of the most dominant kingdom, “bacteria”. The resulting gene feature file was later used during the quantitation of gene expression. The predicted amino acid sequence for each ORF was used to determine Kyoto Encyclopedia of Genes and Genomes (KEGG) orthology (KO) annotations (www.kegg.jp) [28, 29]. Predicted amino acid sequences were searched against the Swiss-Prot database [30] using DIAMOND in blastp mode [31]. KO annotations were determined for each predicted ORF in a consensus fashion (i.e. agreement of >50% of matched references) using KOs reported in the Uniprot ID mapping database [32]. Higher-level KEGG summaries were also generated for KEGG pathway and module annotations as well as BRITE levels 1–3.

### Quantification of ORF expression

Active gene expression in sediment was quantified by comparing the abundance of RNA transcripts relative to that of DNA from the metagenomic assembly. Quantitative information for Prokka-annotated ORFs was determined by mapping raw Illumina reads for both the DNA and RNA samples to contigs with BWA-MEM v0.7.15 [26] using the default parameters -k 19, -w 100, -d 100, -r 1.5, -y 20, -c 500, -D 0.50, -W 0, and -m 50. Raw read counts (i.e., expression levels) of each ORF in each sample were quantified using FeatureCounts [33] using default parameters, and a gene feature file (GFF) was created during annotation of the contigs via Prokka. Genes were filtered to retain those with a total of 100 counts across all samples and had at least 10 counts in three or more samples. Data were normalized using counts per million normalization with the total aligned counts for each sample as the sample size.

Regarding KO analyses, normalized counts for each annotated KO were summed across all annotated ORFs belonging to a particular pathway, module, or BRITE category. First, data were filtered to retain individual KOs with a total of ≥100 counts across all samples and ten counts in at least three samples. Pathways, module, and BRITE summaries were subsequently filtered to have a total of ≥1000 sequence counts across all samples and ten counts in at least three samples. KO count data were normalized using counts per million with the total aligned counts for each sample as the sample size.

### Statistical comparison of microbial communities, DNA, and RNA

The alpha diversity of read-based taxonomic results was assessed via the Shannon Diversity Index [34] by 1) rarifying kingdom-specific tables to depths based on total sequence counts, 2) generating taxonomic summaries for each rarified/sub-sample table, 3) evaluating each summary separately, and 4) testing for statistically significant differences via the Kruskal-Wallis test [35]. Beta diversity was evaluated via Bray-Curtis measure of dissimilarity [36] using default parameters in R in the vegan library [37]. Prior to analysis, data were log_10_(x+1) transformed and resulting dissimilarity indices were modelled and tested for the significance of season using the Adonis test. Heat maps and hierarchal clusters were generated in Partek Flow v8.0 using the following, respectively: 1) normalized counts of taxa from the metagenome and predicted open reading frames (ORFs) across samples and 2) the Euclidean dissimilarity index and average linkage method to cluster similar expression patterns and taxon abundances.

### Differential analysis of ORF expression data

Differential expression statistics (log_2_fold-changes and p-values) were computed for each Prokka-annotated ORF [27], Kyoto Encyclopedia of Genes and Genomes (KEGG [28]; www.kegg.jp) summary, and KEGG orthology (KO) using normalized count data for sediment DNA (abundance) and RNA (expression) from each taxonomic group (i.e., bacteria, archaea, and fungi). Using edgeR [38], expression data were fit to a linear model, assuming a negative binomial distribution, that included season (i.e., winter versus summer), molecule type (i.e., RNA versus DNA), as well as the interaction of season and molecule type (p-interaction). Significance was determined by performing a pairwise comparison tests of season within and between each data type and p-values were FDR-corrected [24]. A p-interaction ≤ 0.05 indicated significant differential gene expression between seasons based on the interaction of season and molecule-type count data and not solely a change in season or molecule-type. Data were then further filtered by an FDR-corrected p-value (q-winter/summer RNA-value) ≤ 0.05 associated with the difference between winter versus summer RNA transcript levels.

Increases or decreases in transcript abundance relative to that of taxonomically unannotated DNA were referred to as differentially abundant in the winter or summer, respectively, if the following criteria was met: p-interaction value was ≤0.05 followed by a q-winter/summer RNA value ≤ 0.05. We also defined the differential expression of KOs as an increase or decrease in the expression of an orthologous gene function relative to that of quantitated sequence counts for the respective orthologues in winter or summer DNA samples, respectively, such that p-season ≤ 0.05. A stringent pairwise p-value was not used in this instance in order to get an idea of gene expression of entire KEGG pathways. However, significantly differentially expressed KOs met the following criteria: p-interaction ≤ 0.05 in combination with a q-winter/summer RNA ≤ 0.05, respectively.

### Analysis of genes involved in natural product biosynthesis, metal resistance, and antibiotic resistance

Contigs were mined for secondary metabolite biosynthetic gene clusters (BGCs) in the bacterial and fungal version of antiSMASH 5.0 [39]. Default parameters and the following features were used to identify BGCs: knowncluster blast, subcluster blast, and active site finder. Annotated BGCs were then filtered such that there were a total of ≥100 counts across all samples and ≥10 counts in at least three samples. Raw counts corresponding to Prokka-annotated ORFs relative to those of DNA were then filtered to determine differential gene expression, such that p-interaction followed by q-winter/summer RNA ≤ 0.05. The BacMet database was used to mine DNA and RNA for experimentally validated metal resistance genes (MRGs) [40]. The raw counts of ORFs were filtered in the same manner as BGCs. Gradient plots were generated in Partek Flow v8.0 for differentially expressed BGCs and those co-expressed with MRGs. Contigs were also mined for antibiotic resistance genes that were within close proximity or colocalized with BGCs using the Antibiotic Resistant Target Seeker (ARTS) version 2 [41] using default parameters. Duplication and BCG proximity, resistance model screens, and genomes that mapped to the following phyla were selected: Actinobacteria and Alphaproteobacteria.

### Data sharing and nucleotide accession numbers

Raw sequence data and metadata files were submitted in the Sequence Read Archive of the National Center for Biotechnology Information (BioProject identifier, PRJNA540505). Raw data used for all analyses have been deposited in Figshare; DOI: 10.6084/m9.figshare.c.4864863. URL– https://doi.org/10.6084/m9.figshare.c.4864863). See reference [42] for additional data analysis.

## Results and discussion

### Physicochemical characterization

The physicochemical properties of all samples varied between seasons (S1 Table). The water temperature was -0.36°C in January (winter) and 16.4°C in July (summer), with a pH of 3.86 and 3.59, respectively. The sediment pH was within the pH range of water, but more acidic in winter (pH 3.56) than summer (pH 3.78), possibly due to how the sediment accumulated protons [43], reducing their dissociation rates. While there was variable pH among samples from different seasons, more data has to be collected to evaluate the significance of this difference. High redox potentials (423–451 mV) were measured, indicating that EB-90M water was oxidized (aerobic environments have redox potentials ≥-100 mV; [44]). Water sulfate levels (95–126 mg/L) were within EPA-recommended concentrations (<250 mg/L) and consistent with former Ely Brook geological studies [3] but less than those reported in other ARD studies [45]. Most nutrients, including nitrate and nitrite (<0.02 mg/L), total Kjeldahl nitrogen (<0.7 mg/L), and reactive and total phosphorus (<0.15 mg/L) in water were below the detection limit. Low levels of total and dissolved organic carbon (1.4–3.1 mg/L) were also detected in water, which is characteristic of ARD due to competition between species and the inability of the environment to retain nutrients [46, 47].

High metal concentrations were detected in all EB-90M samples. The most abundant elements in water were Mg, Al, and Fe (3.07–5.89 mg/L; Table 1), and the amounts of total and dissolved elements were the same across water samples. Silica (SiO_2_, 49%), Fe_2_O_3_ (27%), and Al_2_O_3_ (13%) were the major components of sediment (Table 2), which is also supported by the high levels of Si, Al, and Fe detected by ICAP-MS (Table 1). The weight percent of Fe and Al has increased by 17% and 4%, respectively, since the EPA last analyzed the geochemical properties of EB-90M sediment in 2006 [4, 48], underscoring the long-term detrimental effects of ARD on un-remediated sites.

**Table 1:** Chemical composition of samples.

Sample	Na	Mg	Al	Cr	Mn	Fe	Co	Ni	Cu	Zn	As	Cd	Ba	Pb	Sb
**July water (D)**	1.73 (0.04)	4.17 (0.08)	4.93 (0.12)	<0.01	0.404 (0.003)	5.22 (0.15)	0.0882 (0.0022)	0.0243 (0.0002)	1.85 (0.01)	0.369 (0.006)	<0.01	<0.01	0.0170 (0.0002)	<0.01	<0.01
**July water (T)**	1.76 (0.02)	4.24 (0.08)	5.03 (0.05)	<0.01	0.403 (0.004)	5.62 (0.06)	0.0918 (0.0010)	0.0253 (0.0003)	1.87 (0.02)	0.360 (0.003)	<0.01	<0.01	0.0172 (0.0002)	<0.01	<0.01
**January water (D)**	1.33 (0.01)	3.19 (0.02)	4.31 (0.25)	<0.01	0.286 (0.001)	5.80 (0.01)	0.101 (0.001)	0.0230 (0.0002)	2.27 (0.01)	0.321 (0.004)	<0.01	<0.01	0.0119 (0.0002)	<0.01	<0.01
**January water (T)**	1.31 (0.02)	3.07 (0.06)	4.33 (0.08)	<0.01	0.278 (0.003)	5.89 (0.08)	0.0982 (0.0014)	0.0223 (0.0004)	2.22 (0.00)	0.305 (0.004)	<0.01	<0.01	0.0113 (0.0001)	<0.01	<0.01
**July sediment**	14.3 × 10^3^ *	11.3 × 10^3^ *	66.1 × 10^3^ *	137 (1)	852*	194 × 10^3^ *	20.2 (0.2)	28.5 (0.3)	2.21 × 10^3^ (26)	323 (5)	2.99 (0.21)	0.195 (0.027)	471 (1)	54.6 (0.2)	4.23 (0.09)
**January sediment**	17.4 × 10^3^ *	10.7 × 10^3^ *	64.3 × 10^3^ *	118 (3)	1.04 × 10^3^ *	181 × 10^3^ *	15.2 (0. 2)	25.6 (0.6)	1.90 × 10^3^ (32)	609 (10)	2.44 (0.38)	0.211 (0.045)	806 (4)	52.9 (0.3)	6.49 (0.65)

Selected elemental analysis for water and sediment at EB-90M collected in July 2017 and January 2018 in mg/L and mg/kg, respectively. (D) and (T) represent dissolved and total elements in water samples, respectively. Values with an asterisk (*) were determined by X-ray fluorescence, a more accurate method for the analysis of selected elements in sediment. All other values were determined in triplicate by ICAP-MS.

**Table 2:** Chemical composition of EB-90M sediment in weight percentages.

Chemical composition, wt % (mg/kg)	Summer sediment	Winter sediment
Na_2_O	1.88 (14.3 × 10^3^ Na)	2.11 (17.4 × 10^3^ Na)
MgO	1.98 (11.3 × 10^3^ Mg)	1.85 (10.7 × 10^3^ Mg)
Al_2_O_3_	12.9 (66.1 × 10^3^ Al)	12.2 (64.3 × 10^3^ Al)
SiO_2_	48.0 (230 × 10^3^ Si)	51.7 (241 × 10^3^ Si)
P_2_O_5_	0.156 (697 P)	0.168 (748 P)
K_2_O	2.17 (17.5 × 10^3^ K)	2.25 (18.7 × 10^3^ K)
CaO	1.56 (122 × 10^3^ Ca)	1.93 (137 × 10^3^ Ca)
TiO_2_	0.763 (4.68 × 10^3^ Ti)	0.803 (4.87 × 10^3^ Ti)
MnO	0.0870 (852 Mn)	0.132 (1.04 × 10^3^ Mn)
Fe_2_O_3_	28.4 (194 × 10^3^ Fe)	26.0 (181 × 10^3^ Fe)

Values in the parentheses represent concentrations of selected elements in units of mg/kg.

### Microbial community diversity and composition

**Fig 2:**
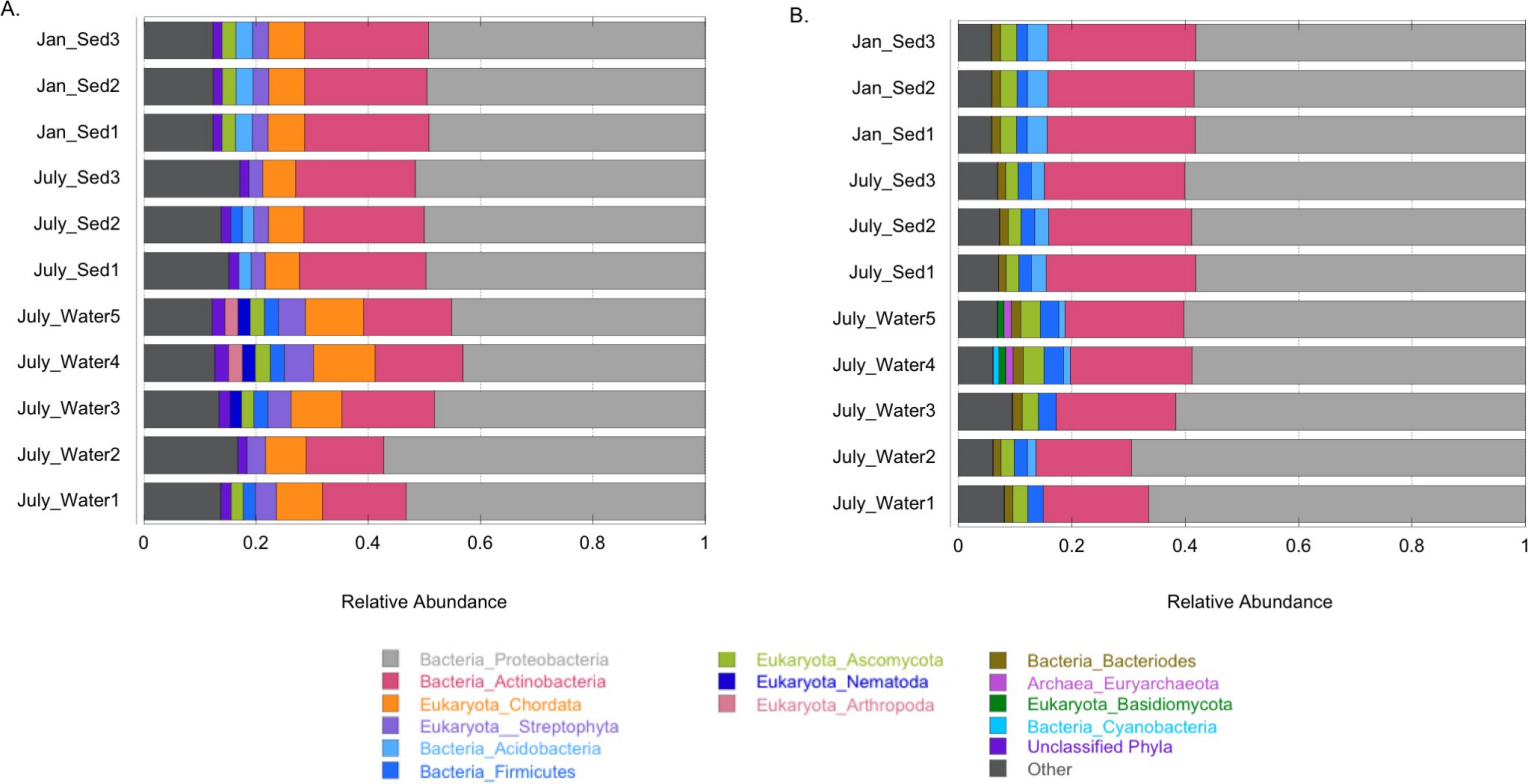
Taxonomic annotation. Seasonal profile of A) all phyla and B) microbial phyla across all samples at EB-90M. Taxa are annotated by superkingdom followed by phylum. “Other” represents phyla that are less than 2% and 1% percent of the data for all organisms and microorganisms, respectively.

Approximately 25% of paired-end reads from 11 out of 16 samples (S2 Table) were taxonomically annotated via Centrifuge and 141 phyla [42], including candidate phyla, were detected across all domains. Data from winter water samples were excluded, as there was considerably lower sequencing coverage due to low DNA yields. All taxonomic annotation data from Centrifuge are available at https://doi.org/10.6084/m9.figshare.c.4864863 (FigShare 1). Of the annotated taxa, *Bacteria* dominated the entire EB-90M community followed by *Eukaryota* and *Archaea* (Fig 2A), respectively, and viruses were also detected. Proteobacteria (50 ± 4%) was the most dominant phylum followed by Actinobacteria (19 ± 4%), Chordata (7.6 ± 0.2%), unclassified sequences (19 ± 2%), and Streptophyta (3.4 ± 1%). When only considering microorganisms, Proteobacteria represented 61 ± 4% of the community followed by 23 ± 3% Actinobacteria (Fig 2B). Proteobacteria commonly dominate ARD [49, 50] due to their metabolic plasticity [51] and they include iron and sulfur oxidizers that grow under metal-rich and less-restrictive pH conditions. Similarly, Actinobacteria have been reported in other ARD environments, including 90 microbial communities in a copper tailing impoundment in Anhui Province, China [50, 52, 53]. Both Proteobacteria and Actinobacteria thrive in diverse sediments and have evolved mechanisms to inhabit metal-rich environments [54].

Shannon diversity indices (H) of taxa were similar for species of bacteria (H = 6.8–7.0), archaea (H = 4.0–4.9), and fungi (H = 5.4–5.5) regardless of sample type (S3–S5 Tables) and indicated greater bacterial diversity. The beta diversity of bacteria, archaea, and eukaryota in samples was significant between summer water and sediment (p < 0.05). However, there was no significant dissimilarity between sediment from different seasons (S6–S8 Tables). Non-metric multidimensional scaling (NMDS) plots and principal component analyses (PCAs) of DNA in water and sediment also showed no clear difference at the genus level between taxa in winter and summer sediment [42]. Season explained 66–92% of the dissimilarity of species between sediment, but the p-value was 0.1 at an alpha level of 0.05 (S6–S8 Tables). Thus, more samples need to be evaluated to confirm this dissimilarity, as season can impact ARD taxonomic diversity due to changes in temperature, pH, and metal concentrations [55–57]. Nevertheless, the beta diversity of organisms in summer sediment and water differed at all taxonomic levels (p < 0.05), with high variation (70–87%) based on sample type (S9–S11 Tables).

**Fig 3:**
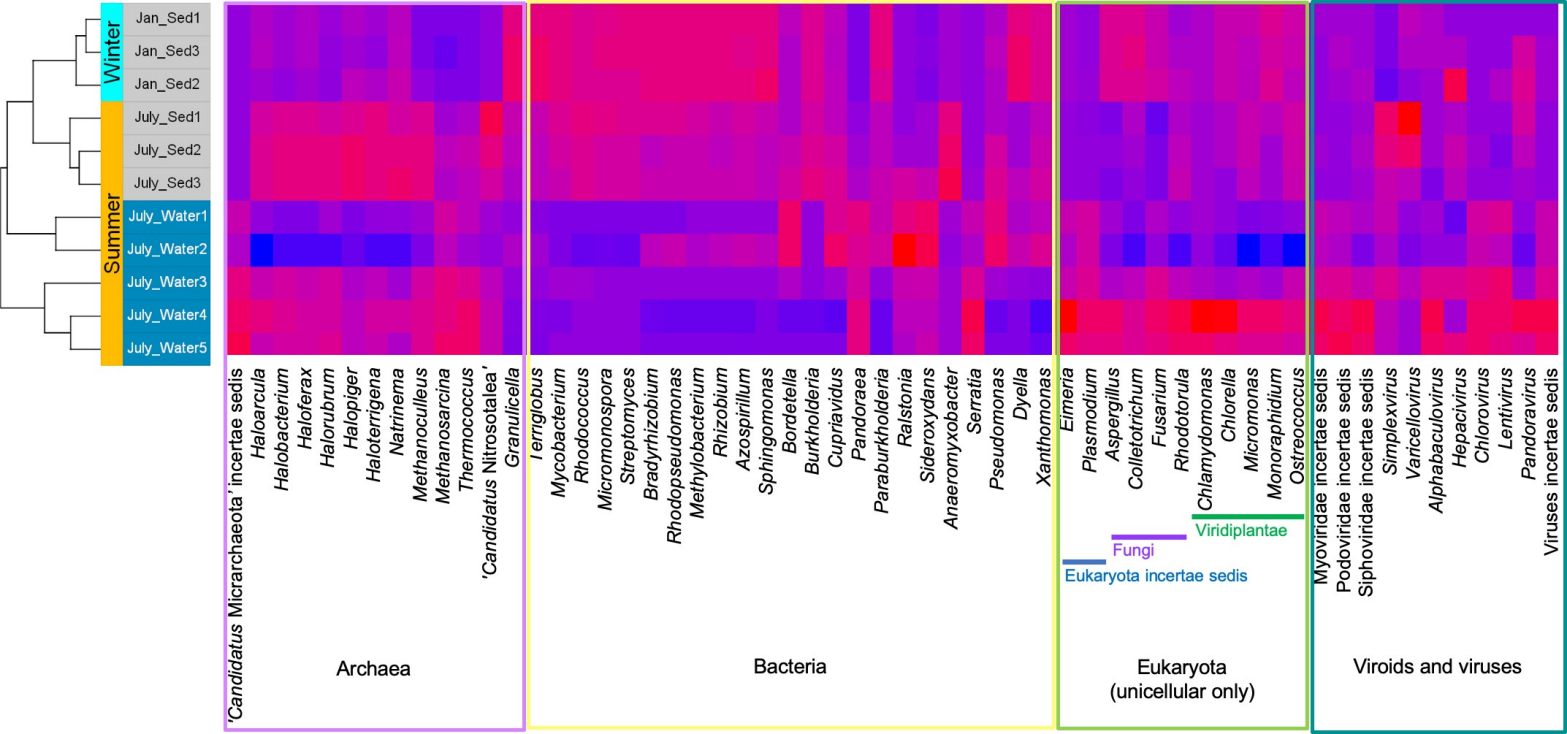
Seasonal microbial diversity. Relative abundances of the most abundant genera of archaea (top twelve), microbial eukaryota (top eleven), and viroid/viruses (top eleven) as well as the 24 most abundant genera for bacteria per sample. The most abundant taxa per sample varied within each grouping (archaea, eukaryota, viroid/viruses, and bacteria). Red and blue represent high and low abundance, respectively. Incertae sedis corresponds to a taxonomic group with unknown broader relationships to other taxa.

*Pseudomonas* (2.6–3.3%), *Bradyrhizobium* (1.7–4.1%), and *Streptomyces* (2.9–5.0%) were the most abundantly annotated microbial genera in all samples (Fig 3). Although not consistent with our hypothesis, acid-tolerant bacteria from these genera have been isolated from other mines [17, 58–64], producing nutrients and mediating the flux of metal ions [65, 66]. Species of *Leptospirillum*, *Acidithiobacillus*, *Acidiphilium*, and *Thiobacillus* were also present in the metagenome but at significantly lower abundance (<0.7%). Considering not all paired-end reads were taxonomically annotated, other sulfur and iron oxidizers may dominate this site.

Most microorganisms were differentially abundant between seasons. For example, of the 1177 annotated bacterial genera, 660 were differentially represented between seasons (p-season < 0.05). Data for the differential analyses of annotated bacteria, archaea, and fungi are available at https://doi.org/10.6084/m9.figshare.c.4864863 (FigShare 2). *Bradyrhizobium*, nitrogen-fixing plant endophytes, were slightly more abundant in the summer regardless of sample type (p-season < 0.05). Archaea were also present but less abundant, as Euryarchaeota represented 1.3 ± 0.03% of the observed microbial phyla (Fig 2B). Euryarchaeota have also been identified in low abundance in the Baiyin open-pit copper mine in China [18]. The most abundant archaeal genus was ‘*Candidatus* Nitrosotalea’, an acidophile involved in nitrification, oxidizing ammonia to nitrite in acidic sediment [67]. This genus was also more prevalent in the summer regardless of sample type (Fig 3). Of the 93 annotated archaeal genera, 55 were differentially represented between seasons (p-season < 0.05).

Chordata was the most abundant eukaryotic phylum (Fig 2A) in the EB-90M metagenome. Within the eukaryotic microbial community, the fungi Ascomycota followed by Basidiomycota were the most abundant eukaryotic phyla (Fig 2B). The following genera were the most represented: *Aspergillus*, *Rhodotorula*, and *Colletotrichum*, with *Rhototorula* being slightly more abundant in the summer (p-season < 0.05) (Fig 3). Ascomycota have been found at mining sites [68], particularly in biofilms at the Richmond Mine at Iron Mountain [69–71], where fungal hyphae provide a surface for symbionts to attach to pyrite sediment [72, 73]. Furthermore, several phyla were algae that inhabit ARD or metal-rich environments (i.e., Bacillariophyta [74], Xanthophyceae [75], and Euglenida [76]). However, all subsequent analyses focused on prokaryotes and fungi, the largest population of unicellular eukaryotes in this dataset. Of 174 fungal genera identified, 89 were differentially represented between seasons (p-season < 0.05).

### Seasonal metabolic and functional activities of taxa

In addition to seasonal variations among taxa, significant seasonal differences in gene expression were observed at EB-90M based on the relative abundance of predicted ORFs. PCA demonstrated uniform but distinct molecular phenotypes across sample type (DNA) and season (ORFs) [42]. Active gene expression in sediment was quantified by comparing the abundance of Prokka-annotated ORFs (S12 Table) to that of the DNA used to assemble the metagenome (S13 Table). All differentially expressed, functionally annotated ORF data are available at https://doi.org/10.6084/m9.figshare.c.4864863 (FigShare 3). Approximately 104,772 out of 296,476 genes were significantly differentially expressed based on a p-interaction ≤ 0.05. Many predicted ORFs (35,037) had a p-interaction value and an FDR-corrected p-value (q-winter/summer RNA-value) ≤ 0.05, indicating several genes were differentially expressed between seasons, which is consistent with other ARD studies in which physicochemical properties were found to impact gene expression [77].

The predicted functions and relative abundance of RNA transcripts provided insight into the roles of taxa, as many contigs did not align to NCBI “nt” sequences in our assembly-based taxonomic annotation pipeline. In the future, additional taxonomic classifiers should be used to increase the taxonomic annotations of functionally annotated genes. Most differentially abundant transcripts encoded hypothetical proteins. Table 3 lists the top ten differentially expressed annotated genes between seasons as well as their producing taxa. These genes were involved in amino acid and cofactor metabolism, protein synthesis, transport, virulence [78], cell wall homeostasis/organization, nucleotide, carbohydrate, and lipid metabolism, cell signaling, and transcription, which are important for survival and have been reported in ARD [45, 49, 79]. Interestingly, *comEC*, a gene involved in horizontal gene transfer (HGT), was highly expressed in winter. HGT or DNA uptake via cellular membranes is involved in the evolution and adaptation of species, which is largely driven by environment and community composition [80] and likely plays a significant role in adaptation to this harsh environment. Furthermore, most genes in Table 3 were expressed by species of *Bradyrhizobium*, *Streptomyces*, *Aromatoleum*, *Methylococcus*, and ‘*Candidatus* Solibacter’, which are common to polluted environments [81–84]. The most abundant gene in the entire dataset, especially in winter, was *stp*, encoding a spectinomycin tetracycline efflux pump belonging to the major facilitator superfamily [85], which *Acidimicrobium* has been reported to express in wastewater [86]. Efflux pumps aid in cellular detoxification and homeostasis, and their expression can be triggered by heavy metal ions, which are abundant at EB-90M (Tables 1 and 2) [87, 88].

**Table 3:** Seasonal gene expression.

	Protein function, Gene	Biological process	Organism
**Highly expressed in Winter **			
	Malto-oligosyltrehalose trehalohydrolase, *treZ*	Trehalose/glycan biosynthesis, virulence	*Aromatoleum aromaticum*
	tRNA-2-methylthio-*N*(6)-dimethylallyladenosine synthase, *miaB*	tRNA methylation	
	Macrolide export protein, *macA*	Antibiotic resistance	*Methylococcus capsulatus*
	Multidrug resistance protein, *stp*	Regulation of EF-tu, virulence, cell wall synthesis, and multidrug resistance	*Acidimicrobium ferrooxidans*
	dITP/XTP pyrophosphatase, *rdgB*	Purine nucleoside catabolism	
	Toxin, *fitB*	Virulence, stress response	
	Enamidase, *Ena*	Cofactor catabolism	*Bradyrhizobium* sp. S23321
	ComE operon protein, *comEC*	Competence for transformation	
	Putative sugar transferase, *epsL*	Cell wall organization	
	Sensor histidine kinase, *tmoS*	Two component regulatory system	
**Underexpressed in Winter**			
	Leucine-, isoleucine-, valine-, threonine-, and alanine-binding protein, *braC*	Amino acid transport	
	6-Oxocyclohex-1-ene-1-carbonyl-CoA hydrolase, *bamA*	Benzoyl CoA catabolism	
	Putative phosphoserine phosphatase 2, *pspB*	Serine biosynthesis	
	Coenzyme PQQ synthesis protein, *pqqD*	Cofactor biosynthesis	
	UDP-N-acetyl-D-glucosamine 6-dehydrogenase, *wbpA*	Cell wall organization	
	Ferric uptake regulation protein, *fur*	Transcription regulation	*Streptomyces avermitilis*
	Glycerophosphodiester phosphodiesterase, *glpQ*	Glycerol and lipid metabolism	
	ABC transporter permease, *ytrF*	ABC transporter involved in acetoin utilization	
	3-Dehydroquinate dehydratase, *aroQ*	Aromatic amino acid biosynthesis	
	Succinate-acetate/proton symporter, *satP*	Acetate-uptake transporter	Candidatus *Solibacter usitatus*

Top ten Prokka-annotated differentially expressed genes in winter sediment paired with taxonomic annotations. Data were sorted by a p-interaction value ≤ 0.05 followed by q-winter/summer RNA value ≤ 0.05, respectively. All RNA transcripts were at least 8 to 20-fold higher or lower compared to DNA in samples. No taxon was annotated if a contig did not align to sequences in “nt”.

### Functional analysis of metagenome and metatranscriptome

KEGG annotated 1,048,574 protein-coding reads with KOs, providing insight into the ecological and metabolic roles of active taxa in sediment. All KEGG annotation data are available at https://doi.org/10.6084/m9.figshare.c.4864863 (FigShare 4). While the function of most ORFs were unknown, 442,447 annotated ORFs were assigned to 6,997 KOs, which were then assigned to KEGG pathways, BRITE hierarchies, and modules. KEGG reference pathway maps and BRITE reference hierarchies are applicable to any organism by functional orthologs being defined by K numbers, which can be used to reconstruct pathways from explicitly incomplete datasets [89]. The most abundant RNA transcripts were involved in BRITE hierarchies and metabolism [42]. Four hundred and fifteen metabolic pathways were identified and mainly involved in carbohydrate metabolism, energy metabolism, and amino acid metabolism, similar to Prokka-identified ORFs in EB-90M sediment as well as those in other ARD sites [45, 90].

**Fig 4:**
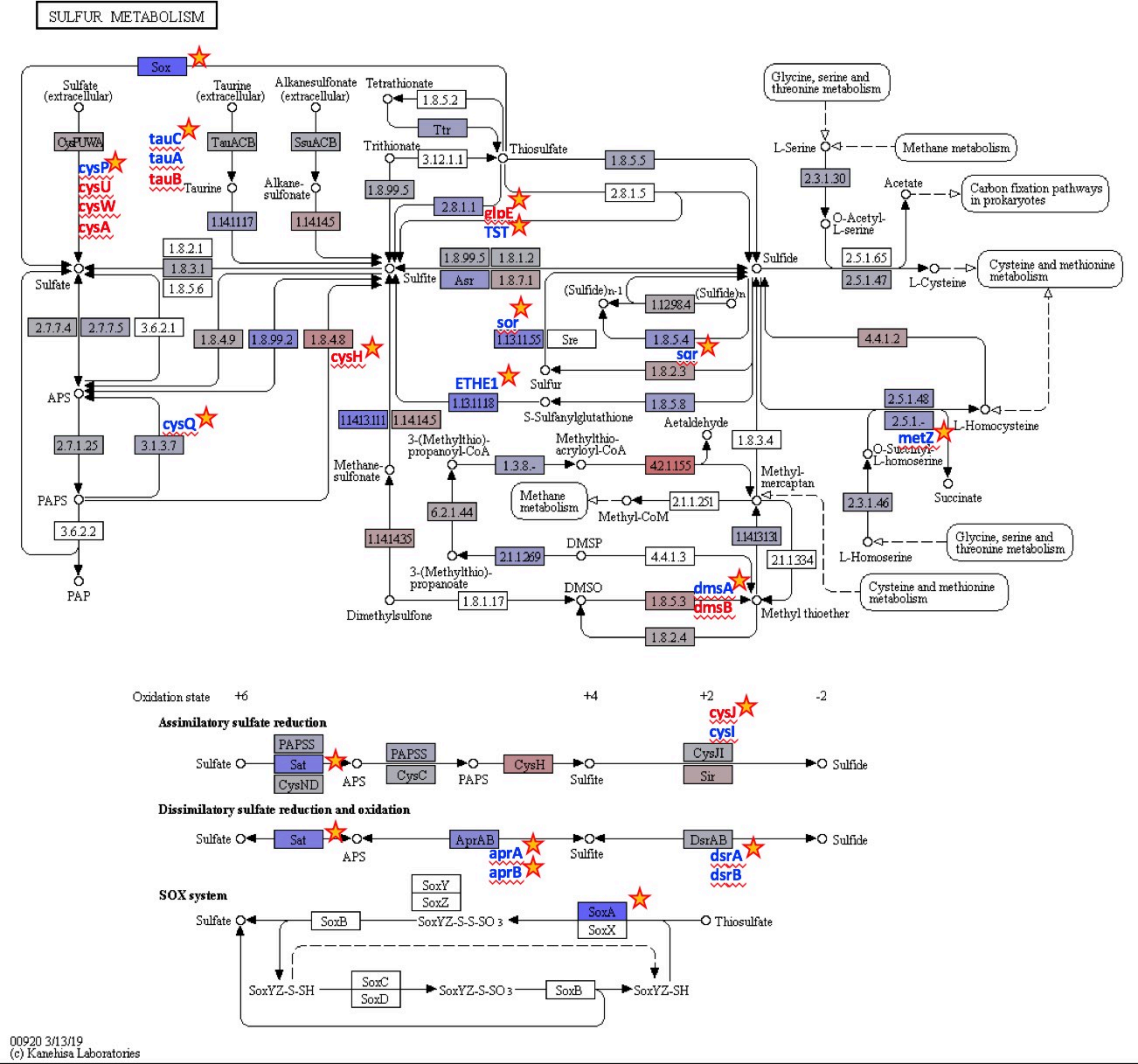
Sulfur metabolism gene expression. Sulfur metabolism KEGG reference pathway map diagram (https://www.kegg.jp/pathway/map00920) with color gradation highlighting KEGG-annotated gene expression that changes between seasons. Blue and red colors denote decreased and increased abundance of RNA transcripts in the winter, respectively. Genes that did not change are light gray and undetected genes are white. Significantly differentially expressed genes are indicated by a star and met the following criteria: p-interaction ≤ 0.05 in combination with a q-winter/summer RNA ≤ 0.05, respectively.

Out of 6,997 KOs, representing annotated ORFs, 2,532 were differentially expressed between seasons based on a p-value related to the significance of season (p-season) < 0.05. In the winter, some of the most differentially expressed KEGG pathways were related to protein digestion and absorption as well as phenazine biosynthesis [42]. While KOs can belong to several pathways, modules, and BRITE hierarchies, most differentially expressed KOs were related to protein families: signaling and cellular processes (BRITE Level 2, 09183). Most transcript levels decreased in winter/increased in summer, possibly due to summer temperatures increasing the metabolic demands of the community, as shown in model ARD biofilms grown at different temperatures [91]. Several pathways, including those involved in sulfur (Fig 4), nitrogen, and carbon metabolism (see FigShare 5–6; https://doi.org/10.6084/m9.figshare.c.4864863), were differentially expressed (p-season < 0.05) [42]. Experimental investigation of these orthologous functions and individual genes is required to confirm the level of gene expression with respect to season.

### Actively expressed genes involved in iron and sulfur cycling

While common iron and sulfur oxidizers did not dominate EB-90M, several ORFs from EB-90M sediment were involved in iron and sulfur cycling. The expression data for differentially expressed KOs involved in iron and sulfur metabolism are available at https://doi.org/10.6084/m9.figshare.c.4864863 (FigShare 7–8). KOs associated with key iron oxidation and reduction genes, such as *cox* and *mtr* genes, were identified. Unique ORFs (584) mapped to KOs encoding *coxABC* genes (K02274, K02275, and K02276) involved in Fe^2+^ oxidation were slightly more expressed in winter. These data are consistent with the presence of Fe^3+^ reducers, such as species of *Granulicella*, in sediment. While a total of 483 transcripts were involved in reducing Fe^3+^ to Fe^2+^, specifically *mtrCAB* (K03585, K07670, and K07654), the KOs were not differentially expressed. However, Prokka-annotated *mtrCAB* ORFs were differentially expressed in the summer. Some acidophiles reduce iron under suboxic conditions [92], are facultative anaerobes (e.g., *Bradyrhizobium*), or inhabit anoxic microzones in sediment where biotic Fe^3+^ reduction occurs.

Several organisms were involved in sulfur cycling, a process involving the movement of sulfur between rocks, water, and organisms. Fig 4 shows the differentially expressed KOs associated with the sulfur metabolism KEGG pathway. KOs (24 out of 104) were differentially expressed in the summer, specifically those involved in dissimilatory sulfate reduction and oxidation pathways, such as *aprBA* (68 ORFs mapped to K00394 and K00395). Organisms expressing these genes could be used for bioremediation by removing excess toxic sulfate from the environment via this reversible pathway [93]. Dissimilatory pathways generate energy and either produce sulfides anaerobically or sulfate aerobically, whereas assimilatory pathways can reduce inorganic sulfate to sulfide to synthesize sulfur-containing amino acids and metabolites in the presence of oxygen. KOs involved in assimilatory sulfate reduction, such as *cysH* (184 ORFs mapped to K00390), were differentially expressed in the winter. Seventy-five *sox* genes (i.e. *soxAB*; K00301, K00302, and K00303) were involved in sulfur oxidation and differentially expressed in the summer. *sox* genes oxidize thiosulfate, a product of metal sulfide dissolution, to a sulfate intermediate to generate energy or reduce carbon. Thus, the *sox* gene may be expressed in species that thrive in warmer temperatures, as other variables, such as pH and metal levels, were similar between seasons. Altogether, these KO data demonstrate that while common iron and sulfur oxidizers did not dominate EB-90M, organisms are actively involved in iron and sulfur cycling.

### Actively expressed genes involved in nitrogen metabolism

The abundance of transcripts involved in nitrogen metabolism is consistent with nitrogen-fixing *Bradyrhizobium* being the most abundant microbial taxa detected at EB-90M. The expression data for all KOs involved in nitrogen metabolism are available at https://doi.org/10.6084/m9.figshare.c.4864863 (FigShare 7–8). Species of *Bradyrhizobium* fix nitrogen in root nodules for plant growth, especially in acidic [94] and pyrite-rich [63] soil. In summer sediment, species of *Bradyrhizobium* differentially expressed the nitrogenase gene *nifH* (K02588; 28 ORFs), a biomarker for nitrogen fixation, the conversion of molecular nitrogen to ammonia [42]. Given that a subset of RNA transcripts annotated as *nifH* lacked taxonomic annotation, *nifH* may also be expressed by plants detected in our dataset, which require more nitrogen for growth at this time of year [95].

Ammonia can also be made from or assimilated via the reduction of nitrate. Assimilatory genes, such as *narB*/1.7.7.2 (K00367) and *NR* (K010534) (52 ORFs in total), which convert nitrate to nitrite, were differentially expressed [42]. Transcripts encoding *narB* (K00367) were differentially abundant in winter, whereas those encoding *NR* (K010534) were differentially abundant in summer. Thus, selected microorganisms along with other plants likely regulate nitrate levels in sediment between seasons. This differential expression may be related to the abundance of nitrogen-fixing bacteria and the abundance of transcripts encoding *nifH*, resulting in the production of excess ammonia in the summer.

### Actively expressed genes involved in carbon metabolism in photosynthetic organisms

Carbon fixation can occur via the Calvin-Benson-Bassham cycle in plants, algae, and phyla of bacteria, such as Cyanobacteria, Chlorobi, Proteobacteria, Firmicutes, Acidobacteria, and Chloroflexi. Some of these bacterial phyla were dominant at EB-90M and reduced the expression of key genes in this cycle, such as *rbcL* and *rbcS* (*RuBisCo*, 4.1.1.39 [42]) as well as *prkB* (2.7.1.19 [42]), in winter likely due to less sunlight [96]. The expression data for all KOs involved in carbon fixation are available at https://doi.org/10.6084/m9.figshare.c.4864863 (FigShare 7–8). In the summer, there were significantly more transcripts of *RuBisCo* (144 ORFs) and *prkB* (51 ORFs), encoding enzymes that fix carbon dioxide to ribulose-1,5-bisphosphate to form 3-phosphoglycerate and phosphorylate ribulose-5-phosphate [97], respectively.

Other carbon fixation pathways were also represented in EB-90M ARD. For example, organisms expressed reductive tricarboxylic acid genes belonging to the C4-dicarboxylic acid pathway [42], which converts carbon dioxide to acetyl-CoA. ORFs (271 of *mdh*, 1.1.1.82) encoding malate dehydrogenase, an enzyme that converts oxaloacetate to malate in this pathway [42], were expressed in the summer to convert oxaloacetate into glucose for energy. Methanogens, prokaryotes that reduce carbon dioxide to produce methane, were also identified based on the expression of the methanogenic genes *mcrA* [98] (five ORFs; K07451) and *mcrB* (11 ORFs; K07452) in both seasons.

### Secondary metabolic pathways

**Fig 5:**
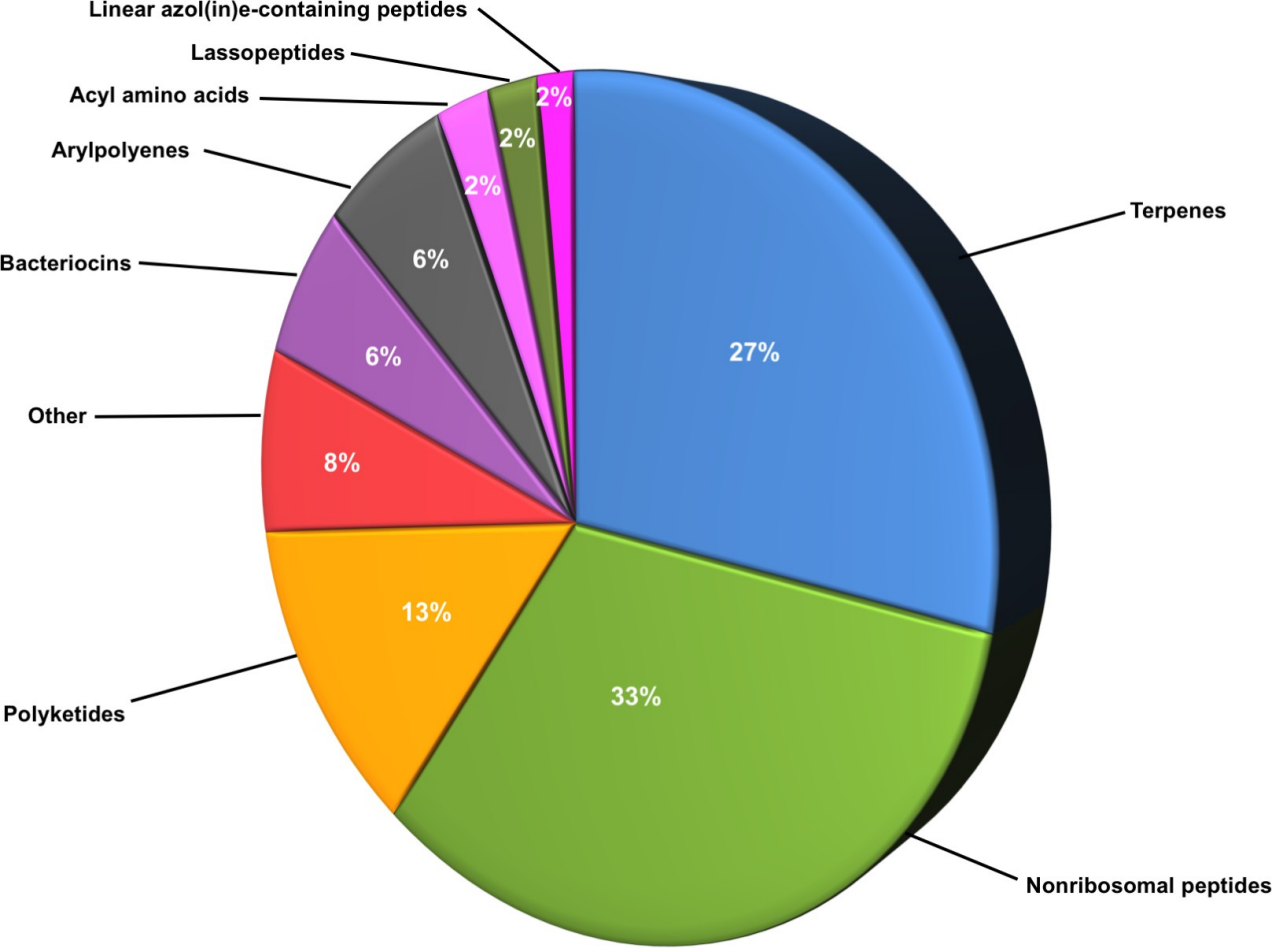
Percentage of genes dedicated to producing various classes of secondary metabolites. Pi chart of antiSMASH-annotated BGCs involved in secondary metabolite production in all EB-90M DNA samples (sediment and water). Classes that represent <2% of 449 BGCs are classified as “Other”.

A total of 1589 BGCs were annotated by antiSMASH 5.0 [39], but only 449 met the filtering criteria [42]. The antiSMASH annotation and associated expression data are available at https://doi.org/10.6084/m9.figshare.c.4864863 (FigShare 9–10). Most BGCs were involved in the biosynthesis of nonribosomal peptides (33%) followed by terpenes (27%) (Fig 5). Since Actinomycetes are present in this dataset, particularly *Streptomyces*, which are prolific producers of secondary metabolites [99], we expected to find BGCs dedicated to secondary metabolism. However, most BGCs were found in contigs without taxonomic annotation. A subset of BGCs is identical to those involved in producing carotenoids, anabaenopeptin NZ 857/nostamide A, rhizomide A–C (cytotoxic [100]), xenotetrapeptide, *n*-acyl alanine, alkyl resorcinol, 1-heptadecene, bicornutin, patellazole (cytotoxic [101]), micromonolactam, geosmin, and phomopsin (tubulin polymerization inhibitor [102]). These data are consistent with our hypothesis that EB-90M can be a source of bioactive metabolites, as there are BGCs involved in producing bioactive compounds. Notably, there is the potential to find more, as many BGC products are unknown.

**Fig 6:**
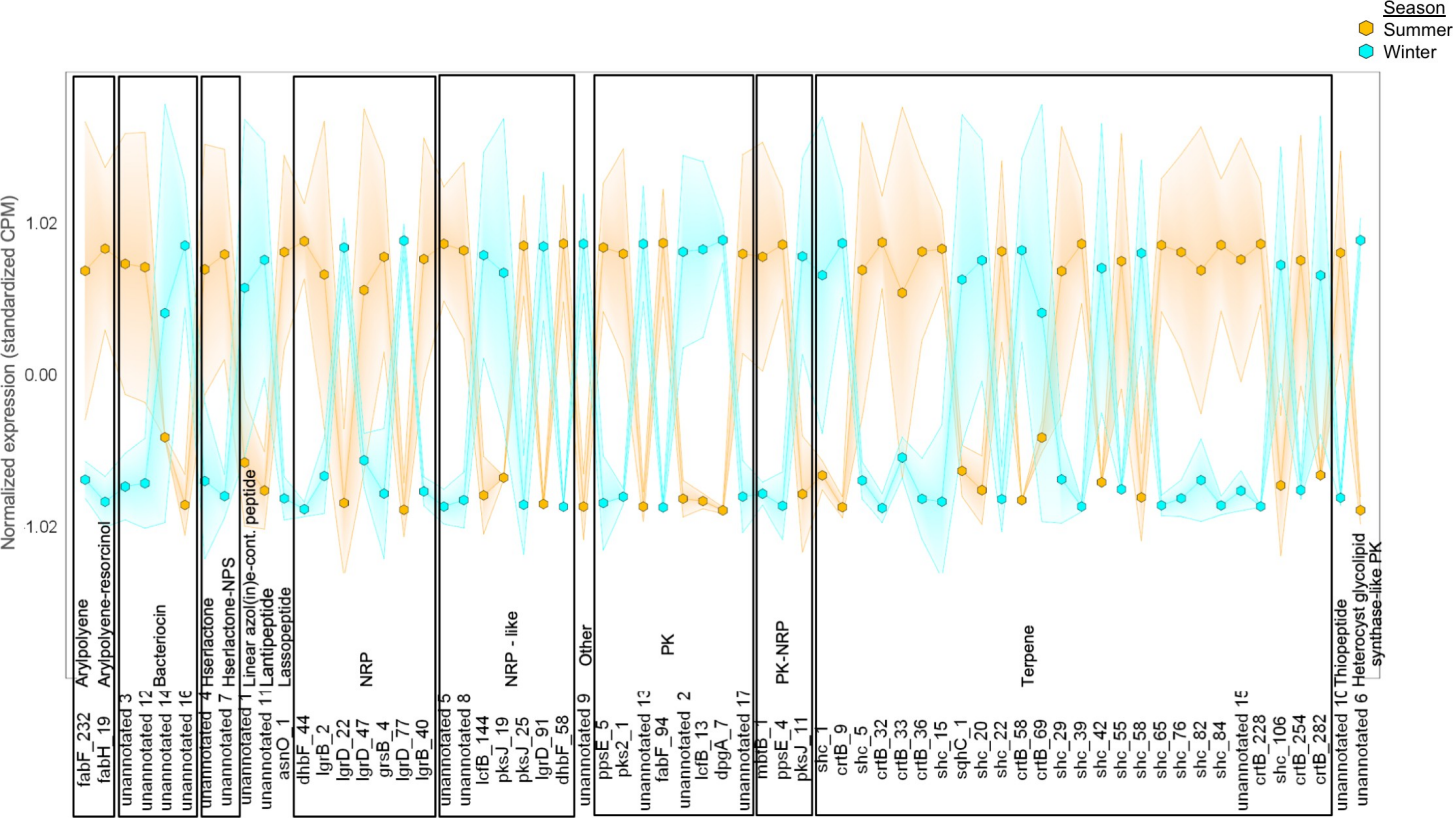
Seasonal expression of terpene BGCs terpene- and NRP-annotated BGCs. Gradient plot demonstrating the expression of BGCs in winter (light blue) and summer (orange) sediment samples. NRP, nonribosomal peptide; PK, polyketide. All data met the following criteria: p-interaction ≤ 0.05 followed by q-winter/summer RNA ≤ 0.05.

Within the metatranscriptomic datasets, 65 out of 449 transcripts encoding genes within BGCs were differentially abundant in summer (39 genes) than winter (26 genes) based on p-interaction and q-winter/summer RNA values < 0.05 (Fig 6). The expression of the phytoene synthase gene, *crtB*, in the summer increased in some organisms and decreased in other organisms (S14 Table), suggesting that selected microorganisms may alter their metabolism based on the presence of other taxa and ecological factors that require specific metabolites. Overall, there were more annotated terpenes and nonribosomal peptides produced in the summer.

### Metal resistance genes (MRGs)

Of the 285 experimentally validated MRGs in the BacMet database, 133 were identified in the metagenome, representing 7,021 unique ORFs out of 161,984. All BacMet annotation and expression data are available at https://doi.org/10.6084/m9.figshare.c.4864863 (FigShare 11). These data are consistent with our hypothesis, as microorganisms conferring metal resistance are commonly found in ARD for the sequestration or chemical conversion of toxic heavy metals. Eight of the 133 BacMet genes were not expressed [42] and 719 of the 7,021 ORFs were differentially expressed in the summer [42]. Mostly transcripts without taxonomic annotation and Proteobacteria of the genus *Burkholderia* conferred heavy metal resistance to Cu, Cd, Co, Zn, Fe, Ag, Pb, Hg, As, Sb, and Ni. Proteobacteria have expressed MRGs in acidic environments [103–106] and been explored for the bioremediation of metal-contaminated environments via efflux pumps, bioabsorption, and transforming metals into less toxic forms [107]. Some of the most differentially expressed MRGs were involved in Cu (*dnaK*) [104], Cu/Te (*actP*) [108], Cd/Co/Zn/Cu (*czcA/B*; *actP)* [105, 109], Cu/Zn/As (*pstA*) [110, 111], Mn/Zn/Fe/Cd/Co (*mntH*) [112], and Zn (*zraR*) [113] resistance. Genes related to As- and Sb-containing compound (*pgpA; acr3*) resistance were differentially expressed, even though Sb and As were nearly undetectable (Table 2). Based on the large number of genes without functional annotation, more proteins with unique mechanisms and metal-binding properties likely exist at EB-90M.

### Resistance mechanisms and secondary metabolism

Metal-rich environments select for MRGs and also co-select for antibiotic resistance genes based on having similar genetic mechanisms [8]. These resistance genes can be used to bioprospect metal-polluted environments for new chemistry and bioactivity. Strategies for prioritizing antibiotic-producing BGCs are based on finding BGCs containing duplicated essential genes, resistance genes, or genetic evidence for HGT [114]. Additionally, there are MRGs encoding proteins (e.g., resistance-nodulation-division family transporters, such as CzcA) that catalyze the efflux of antibiotics and chemotherapeutics [115], and a subset also function as antibiotic resistance genes that can be exploited to find new bioactive compounds [114]. Using the ARTS web server [41], a platform that prioritizes antibiotic-producing BGCs based on these hypotheses, a range of essential (6358–8289) and duplicated (5595–7395) genes, as well as known resistance models (8501), mostly resistance to biotin-lipoyl domains (1585 out of 8501), were annotated [42]. The ARTs annotation data are available at https://doi.org/10.6084/m9.figshare.c.4864863 (FigShare 12). Some duplicated genes colocalized with BGCs, mostly (5–12 out of 258–325) RNA polymerase sigma factor 70 and trigger factor, demonstrating the potential of the ARD microbiome to be a source of antibiotics.

**Fig 7:**
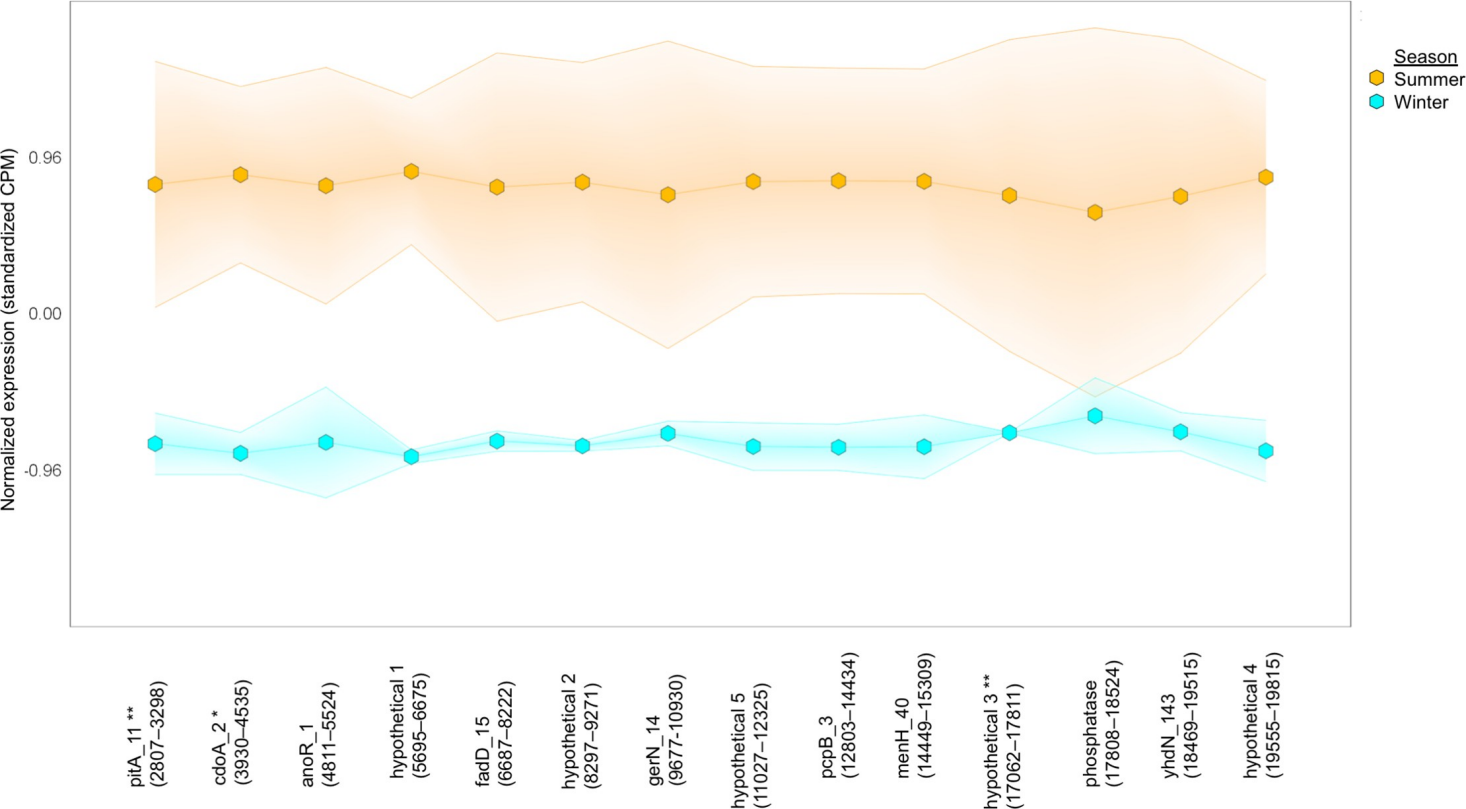
Colocalization and coexpression of metal resistance and secondary metabolite genes. Gradient plot of the differential coexpression of *pitA*, an MRG encoding a phosphate-uptake transport protein, with genes annotated to be involved in the biosynthesis of a secondary metabolite, homoserine lactone-nonribosomal peptide in contig 4689 (20406 nucleotides long), in summer (orange) and winter (blue). All data met the following criteria: p-interaction ≤ 0.05 in combination with a q-winter/summer RNA ≤ 0.05, respectively. Nucleotide positions in contig are shown in parentheses.

MRGs can colocalize with BGCs and play a role in antibiotic resistance [116]. We identified six BGCs that colocalized with BacMet-annotated MRGs on contigs (Fig 7) [42]. Fig 7 shows the colocalization and coexpression of phosphate transport gene *pitA* with a homoserine lactone-nonribosomal peptide-annotated BGC in summer (Fig 7). PitA transports phosphate with other cations, such as toxic metal ions [117], and may be differentially expressed in the summer in response to excess phosphate or metal. Excess metal ions can trigger the production of secondary metabolites, such as metal-binding homoserine lactones [100] or nonribosomal peptides called siderophores [118]. The Cu- and Zn-expressed antibiotic resistance gene *mdtA*, encoding a membrane fusion protein of the multidrug efflux complex MdtABC, also colocalized and coexpressed with the polyketide synthase gene, *ppsE* [42]. Both genes were differentially expressed in the summer; however, both MRG and BGC did not meet q-winter/summer RNA ≤ 0.05 for, but they met a p-winter/summer RNA ≤ 0.05 [42]. A subset of ARTS-annotated resistance genes were also MRGs (i.e., metallopeptidases, HflB [119] and RseP [120]) that colocalized with BGCs. Thus, colocalization and coexpression of MRGs and BGCs could be used to prioritize antibiotic-producing BGCs (e.g., *ppsE*) and find new regulatory mechanisms of secondary metabolism.

## Conclusions

The present study is the first seasonal characterization of a metagenome and metatranscriptome at the Ely Copper Mine Superfund site, providing insight into the microbial community as well as the genes and metabolites they use to adapt to ARD in Ely Brook. Acid-tolerant Proteobacteria were the dominant annotated taxa, varying with season and sample type. Several RNA transcripts were differentially abundant between seasons and the most abundant transcript was involved in antibiotic resistance. KO analysis of Prokka-annotated ORFs identified several differentially expressed genes involved in iron and sulfur, nitrogen, and carbon metabolism, providing insight into seasonal gene function, as taxonomy could not be assigned to many ORFs using our pipeline. Genes involved in metal resistance and secondary metabolism were also annotated and differentially expressed. Future work will involve using additional taxonomic classifiers to assign taxonomy to functionally annotated transcripts as well as experimentally validating the differential expression of selected genes. Importantly, several resistance genes (metal and antibiotic) colocalized with BGCs and, in some instances, were coexpressed, revealing putative antibiotic-producing BGCs and their ecological roles that can be exploited for bioremediation or pharmacological purposes.

## Supporting information

S1 Data (DOCX)

S1 Table Geochemistry of aqueous samples. Physicochemical characteristics of water samples collected in July 28, 2017 and January 14, 2018 at EB-90M. Values with standard errors are an average of three distinct samples collected on the same day. N/A indicates that these parameters were not measured.
(DOCX)

S2 Table Annotation of raw reads. Processing of sequence data with annotated taxonomy. No sequences were trimmed. The annotations made exclude unclassified or unassigned phyla, genera, species, and genes.
(DOCX)

S3 Table Alpha diversity of bacterial taxa in summer water and sediment. Shannon diversity indices assessing alpha diversity of bacterial taxa within July and January sediment and July water samples.
(DOCX)

S4 Table Alpha diversity of archaeal taxa in summer water and sediment. Shannon diversity indices assessing alpha diversity of archaeal taxa within July and January sediment and July water samples.
(DOCX)

S5 Table Alpha diversity of bacterial, archaeal, and fungal taxa in summer water and sediment. Shannon diversity indices assessing alpha diversity of fungal taxa within July and January sediment and July water samples.
(DOCX)

S6 Table Alpha diversity analysis of bacterial taxa within sediment samples and beta diversity across all sediment samples. The alpha diversity analysis of bacterial taxa within sediment samples as well as beta diversity analyses across all sediment samples at different levels of annotation. The Shannon diversity index was determined to assess alpha diversity, and the ADONIS and ANOSIM analyses were used to determine the beta diversity among sediment from January 2018 compared to July 2017. Significance * ≤ 0.05, ** ≤ 0.01, *** ≤ 0.001.
(DOCX)

S7 Table Alpha diversity analysis of archaeal taxa within sediment samples and beta diversity across all sediment samples. The alpha diversity analysis of archaeal taxa within sediment samples as well as beta diversity analyses across all sediment samples at different levels of annotation. The Shannon diversity index was determined to assess alpha diversity, and the ADONIS and ANOSIM analyses were used to determine the beta diversity among sediment from January 2018 compared to July 2017. Significance * ≤ 0.05, ** ≤ 0.01, *** ≤ 0.001.
(DOCX)

S8 Table Alpha diversity analysis of fungal taxa within sediment samples and beta diversity across all sediment samples. The alpha diversity analysis of fungal taxa within sediment samples as well as beta diversity analyses across all sediment samples at different levels of annotation. The Shannon diversity index was determined to assess alpha diversity, and the ADONIS and ANOSIM analyses were used to determine the beta diversity among sediment from January 2018 compared to July 2017. Significance * ≤ 0.05, ** ≤ 0.01, *** ≤ 0.001.
(DOCX)

S9 Table Alpha diversity and beta diversity analyses of bacterial taxa within and across summer water and sediment samples, respectively. Alpha diversity analysis of bacteria as well as the beta diversity analyses across all summer samples (i.e., water and sediment) at different levels of annotation. Significance * ≤ 0.05, ** ≤ 0.01, *** ≤ 0.001.
(DOCX)

S10 Table Alpha diversity and beta diversity analyses of archaeal taxa within and across summer water and sediment samples, respectively. Alpha diversity analysis of archaea as well as the beta diversity analyses across all summer samples (i.e., water and sediment) at different levels of annotation. Significance * ≤ 0.05, ** ≤ 0.01, *** ≤ 0.001.
(DOCX)

S11 Table Alpha diversity and beta diversity analyses of fungal taxa within and across summer water and sediment samples, respectively. Alpha diversity analysis of fungi as well as the beta diversity analyses across all summer samples (i.e., water and sediment) at different levels of annotation. Significance * ≤ 0.05, ** ≤ 0.01, *** ≤ 0.001.
(DOCX)

S12 Table Summary of Prokka-annotated genes. Summary of Prokka-annotated genes in water and sediment samples. All samples had 0 genes that were unassigned due to multi-mapping, duplication, nonjunctions, secondary structure, chimeras, fragment lengths, and mapping quality.
(DOCX)

S13 Table Alignment of raw reads to metagenomic assembly. Alignment statistics for raw reads (i.e., DNA and RNA) to metagenomic assembly in water and sediment samples.
(DOCX)

S14 Table crtB phytoene synthase gene expression. Taxa over- or underexpressing various *crtB* phytoene synthase genes with corresponding log fold change and interaction p-values (Type:Season). Any read that could not be taxonomically annotated does not have taxon listed.
(DOCX)
