# Supplementary material for: Characterization of an acid rock drainage microbiome and transcriptome at the Ely Copper Mine Superfund site
Source: PLoS One. 2020 Aug 12;15(8):e0237599. doi: 10.1371/journal.pone.0237599 (PMC7423320; doi:10.1371/journal.pone.0237599)
Supplement: S1 Data — (DOCX) [file pone.0237599.s001.docx]

**Supporting Information**

*Physicochemical characterization*

Ely Copper Mine is in a continental moist climate with an annual mean temperature of ~6 °C [1]. July is the warmest month of the year with an average temperature of 18.6 °C. January is the coldest month in the year with an average temperature of -9.2 °C. Ely Brook (43°55’9” N, 72°17’11” W) 90 m upstream from the mouth (EB-90M) of the brook, was selected as a study site because the United States Geological Survey (USGS) has collected physicochemical data at this site [2-4] indicating that EB-90M is acidic and its pH fluctuates with seasons. Surface water (July 14^th^, 2017 and July 28^th^, 2017) and sediment (July 28^th^, 2017 and January 14^th^, 2018) were analyzed for different field parameters. Surface water was tested for the following parameters using a handheld, portable Hannah HI98194 multimeter and probes: pH, specific conductance, temperature, and oxidation-reduction potential (ORP). Water samples were analyzed for sulfate, nitrate, nitrite, nitrogen contained in organic substances as well as ammonia and ammonium, alkalinity, phosphorus [reactive (orthophosphate) and total], dissolved organic carbon, and total organic carbon within 48 hours of collection.

Using a HACH DR2400 spectrophotometer and the following HACH (Loveland, CO) reagent sets and methods the amount of sulfate, nitrate, total phosphate, and alkalinity were quantified, respectively, according the manufacturer’s instructions: sulfaver 4 sulfate reagent TNT 864 (method 8051), nitrate TNT 835 (method 10207), and phosphorus, reactive (orthophosphate) and total TNT 843 (method 10209). All samples were analyzed in Middlebury's trace-metal laboratory, with the exception of samples analyzed for nitrate and nitrite, which were analyzed at Endyne (Williston, VT), and total organic carbon (TOC), dissolved organic carbon (DOC), and total Kjehldahl nitrogen were analyzed at Agricultural and Environmental Services Laboratories (Athens, GA).

*Preparation of sediment for ICAP-MS analysis*

Sediment samples underwent complete digestion using a Claisse NeoFluxer with lithium metaborate as a flux. Approximately 0.2 g of sediment was weighed into a crucible with 1.8 g flux and 70 mL of 5% nitric acid was added. Samples were heated to 1050 °C at 0, 10, 20, and 30 rpm using a 21-minute fusion program. The resulting samples were diluted 1000-fold with 5% nitric acid and spiked with an internal standard. See Table S2 for a list of all reference and internal standards used in ICAP-MS analyses.

| Standard reference material | Sample matrix | Certified values of elements |
| --- | --- | --- |
| Traces (1:1 mixture of IV-ICPMS-71A and IV-ICPMS-71B from Inorganic Ventures; Christiansburg, VA) | sediment | 1 ppm Rd, In, and Bi |
| BCR-2 (USGS) | sediment | Al_2_O_3_, 13.5 ± 0.2 wt %  CaO, 7.12 ± 0.11 wt %  Fe_2_O_3_tot, 13.8 ± 0.2 wt %  K_2_O, 1.79 ± 0.05 wt %  MgO, 3.59 ± 0.05 wt %  Na_2_O, 3.16 ± 0.11 wt %  P_2_O_5_, 0.35 ± 0.02 wt %  SiO_2_, 54.1 ± 0.8 wt %  TiO_2_, 2.26 ± 0.05 wt % |
| 2008ISS (Inorganic Ventures; Christiansburg, VA) | water | Bi (20.01 ± 0.11 ppb), Sc (20.01 ± 0.13 ppb), Y (20.01 ± 0.08 ppb) |
| 1643f (NIST; Gaithersburg, MD) | water | Al (133.8 ± 1.2 ppm), Sb (55.45 ± 0.40 ppm), As (57.42 ± 0.38 ppm), Ba (518.2 ± 7.3 ppm), Be (13.67 ± 0.12 ppm), Bi (12.62 ± 0.11 ppm), B (152.3 ± 6.6 ppm), Cd (5.89 ± 0.13 ppm), Ca (29,430 ± 330 ppm), Cr (18.50 ± 0.10 ppm), Co (25.30 ± 0.17 ppm), Cu (21.66 ± 0.71 ppm), Fe (193.44 ± 0.78 ppm), Pb (18.488 ± 0.084 ppm), Li (16.59 ± 0.35 ppm), Mg (7,454 ± 60 ppm), Mn (37.14 ± 0.60 ppm), Mo (115.3 ± 1.7 ppm), Ni (59.8 ± 1.4 ppm), K (1,932.6 ± 9.4 ppm), Rb (12.64 ± 0.13 ppm), Se (11.700 ± 0.081 ppm), Ag (0.9703 ± 0.0055 ppm), Na (18,830 ± 250 ppm), Sr (314 ± 19 ppm), Te (0.9770 ± 0.0084 ppm), Tl (6.892 ± 0.035 ppm), V (36.07 ± 0.28 ppm), Zn (74.4 ± 1.7 ppm) |

Table S1. References used to analyze water or sediment by ICP-MS.

*ICAP-MS analysis of sediment and water*

A Thermofisher inductively-coupled argon plasma mass spectrometry (ICAP-MS) was used to test sediment samples for solid-phase elemental concentrations, and water samples for dissolved and total trace metal concentrations using internal and external standards. The instrument was tuned using the THERMO-5A (Inorganic Ventures, VA) multielement standard according to the manufacturer’s procedures. Performance was verified using the THERMO-4AREV multielement standard to ensure instrument stability (<5%). Helium was used as a collision gas at a rate of 4.9 ml/min. The following elements had dwell times of 0.01 s: Sc, Y, In, Sn, Ba, Tb, and Bi. All other detected elements had dwell times of 0.05 s. The estimated total run time for each sample was 13s 800 ms.

For water analysis, the 2008ISS multielement standard (Inorganic Ventures, VA) was used as an internal reference for all samples at a concentration of 100 ppb to correct for instrument drift. All samples including the standards were spiked with the internal standard. The following five standards were used: 1) a 5% nitric acid solution containing 10.1 ppm Na, Mg, Ca, K, Al, Si, and Fe as well as 100 ppb IV71A, IV71B, and 2008ISS (Sc, Y, Bi, In, and Tb); 2) a 5% nitric acid solution containing 5.05 ppm Na, Mg, Ca, K, Al, Si, and Fe, 50 ppb IV71A and IV71B, and 100 ppb 2008ISS (Sc, Y, Bi, In, and Tb); 3) a 5% nitric acid solution containing 1.01 ppm Na, Mg, Ca, K, Al, Si, and Fe, 10 ppb IV71A and IV1B, and 100 ppb 2008ISS (Sc, Y, Bi, In, and Tb); 4) a 5% nitric acid solution containing 101 ppb Na, Mg, Ca, K, Al, Si, and Fe, 1 ppb IV71A and IV71B, and 100 ppb 2008ISS (Sc, Y, Bi, In, and Tb); and 5) a 5% nitric acid solution containing 100 ppb 2008ISS (Sc, Y, Bi, In, and Tb). The NIST standard reference material 1643f was used to ensure that the instrument was calibrated within 15% per element. We report only estimated values for Ca and Al (see Table S2) due to the 1) Ca level in 1643f being three times more concentrated than the most concentrated standard used to make the standard curves but the value was within 15% of the reported Ca concentration in 1643; and 2) trace amounts of Al contaminating standards containing low concentrations of Al precluding our verification of being within 15% of the 1643f reference, which also contained low concentrations of Al, but our actual samples had higher concentrations of aluminum and should be less affected by this. All standard curves had R^2^ values of 0.99. All other elements analyzed are reported in Table S3.

|  | July 2017 (mg/L) | January 2018 (mg/L) |
| --- | --- | --- |
| Dissolved metal | 496 Ca, 823 Al | 342 Ca, 631 Al |
| Total metal | 447 Ca, 734 Al | 319 Ca, 592 Al |

Table S2. Estimate concentrations of calcium and aluminum ions in water samples.

For ICAP-MS analyses of sediment, samples were digested using a Claisse NeoFluxer with lithium metaborate as a flux. The resulting samples were diluted 1000-fold, spiked with an internal standard (1 ppm Rh, In, and Bi) and analyzed using internal and external standards (BCR-2) (Table 1). Five calibration standards of IV-71a and IV71b (0­­–50 μg/L) spiked with internal standards were also prepared. X-ray fluorescence was also used to accurately quantify elements in sediment using the BCR-2 standard.

*X-ray fluorescence analyses*

For elemental analysis, sediment was dried, sieved, ground, homogenized, ignited, and stored dry prior to analysis. The major ions in sediment were determined via fused-disk. Fused-disk analyses were performed on a Thermo Scientific ARL QUANT’X X-ray Fluorescence Spectrometer using the Basalt, Columbia River (BCR-2) USGS geochemical standard (Table S2).

Figure S1. Flow chart representing the workflow for the metagenomic taxonomic analysis of data. Document shapes (i.e., shapes with the curvy bottoms) represent main files/output. Rectangles represent processes/scripts/pipelines. Parallelograms represent intermediate files and cylinders represent references.

Figure S2. Flow chart representing the workflow for the metatranscriptomic taxonomic analysis of data. Document shapes (i.e., shapes with the curvy bottoms) represent main files/output. Rectangles represent processes/scripts/pipelines. Parallelograms represent intermediate files and cylinders represent references. Ovals represent filtering steps.

*DNA and RNA extraction and library construction*

Total DNA was extracted from water (≥1 L filtered) and wet sediment samples (0.25 g) collected on July 28^th^, 2017 and January 14^th^, 2018 using the DNeasy PowerWater Sterivex^®^ and PowerSoil^®^ DNA Isolation kits (Mo Bio Laboratories, Inc.; Carlsbad, CA) according to the manufacturer’s instructions. Extractions were performed in triplicate on the same samples; however, RNA and DNA were extracted from different batches of sediment or water samples collected at the same time on either July 28^th^ 2017 or January 14^th^, 2018 at EB-90M. Total RNA was extracted from sediment samples (2.0 g) using the RNeasy PowerSoil^®^ Total RNA Isolation kit (Mo Bio Laboratories, Inc.; Carlsbad, CA). For RNAseq, microbial ribosomal RNA (rRNA) depletion and library preparation were performed using the ScriptSeq Complete kit for bacteria (Illumina ScriptSeq with Ribo-Zero Bacterial rRNA removal) following the manufacturer's protocol. Due to the limitations of the depletion protocol, some microbial and all fungal rRNAs were not removed and remained in the sequence data. For shotgun sequencing, libraries were prepared using the Nextera XT protocol (Illumina; San Diego, CA) according to the manufacturer’s instructions. A total of 16 DNA (five July 2017 water samples, three July 2017 sediment samples, five January 2018 water samples, and three January 2018 sediment samples) and six RNA samples (three January 2018 and three July 2017 samples from sediment) yielded viable sequence data.

*Metagenomic and metatranscriptomic sequencing*

DNA library sequencing was performed on an Illumina NextSeq500 instrument, employing a mid-output kit, with paired-end 150 base sequencing reads. RNA library sequencing was performed using an Illumina NextSeq500 high-output kit, with paired-end 150 base sequencing reads. Library preparation and sequencing were performed at the University of Illinois at Chicago Sequencing Core (UICSQC). Libraries were pooled in equimolar ratio and sequenced using an Illumina NextSeq500 instrument at UIC, implementing paired-end 2 × 150 base reads. All samples were spiked with 1% phiX and roughly 12 Gb of data (50 M clusters) were produced per sample. A total of 11 DNA (five July 2017 water samples, three July 2017 sediment samples, and three January 2018 sediment samples) and six RNA samples (three January 2018 and three July 2017 samples from sediment) yielded viable sequence data. No viable data was obtained for water samples collected in January. Raw sequence data were not trimmed due to the subsequent annotation pipeline being insensitive to low quality data at edges.

*Other data analysis*

For the differential analyses of Prokka-annotated genes, KEGG-annotated genes, BacMet metal resistance genes, see reference [49] and/or Figshare; DOI: 10.6084/m9.figshare.c.11879094. URL – https://doi.org/10.6084/m9.figshare.c. 11879094). These references also contain data pertaining to the annotation secondary metabolic biosynthetic genes, BacMet metal resistance genes, and antibiotic resistance genes as well as their colocalization and coexpression.

**References**

1. USDA. Vershire series: United States Department of Agriculture; 2011 [cited 2018]. Available from: <https://soilseries.sc.egov.usda.gov/OSD_Docs/V/VERSHIRE.html>.

2. Seal RR, Kiah RG, Piatak NM, Besser JM, Coles JF, Hammarstrom JM, et al. Aquatic assessment of the Ely Copper Mine Superfund site, Vershire, Vermont. US Geological Survey, 2010 2328-0328.

3. Kimball BE, Foster AL, Seal RR, Piatak NM, Webb SM, Hammarstrom JM. Copper speciation in variably toxic sediments at the Ely Copper Mine, Vermont, United States. Environ Sci Technol. 2016;50(3):1126-36.

4. Piatak NM, Hammarstrom JM, Seal RR, Briggs PH, Meier AL, Muzik TL, et al. Geochemical characterization of mine waste at the Ely Copper Mine superfund site, Orange County, Vermont. 2004 Contract No.: 2004-1248.
