# Supplementary material for: Characterization of an acid rock drainage microbiome and transcriptome at the Ely Copper Mine Superfund site
Source: PLoS One. 2020 Aug 12;15(8):e0237599. doi: 10.1371/journal.pone.0237599 (PMC7423320; doi:10.1371/journal.pone.0237599)
Supplement: S1 Table — Physicochemical characteristics of water samples collected in July 28, 2017 and January 14, 2018 at EB-90M. Values with standard errors are an average of three distinct samples collected on the same day. N/A indicates that these parameters were not measured. (DOCX) [file pone.0237599.s002.docx]

| Sample | pH | Water temperature  (°C) | Air temperature  (°C) | [Sulfate] (mg/L) | Total organic carbon (mg/L) | Total dissolved carbon (mg/L) | Oxidative reduction potential (mV) | Conductivity (μS/cm) |
| --- | --- | --- | --- | --- | --- | --- | --- | --- |
| July 2017 water | 3.36 | 16 | 21 | 126 | 3.13 ± 0.22 | 3.13 ± 0.22 | 423 | 476 |
| January 2018 water | 3.86 | -0.36 | -17 | 95 | 1.95 ± 0.23 | 1.43 ± 0.23 | 451 | 258 |
| July 2017 sediment | 3.78 | N/A | 21 | N/A | N/A | N/A | N/A | N/A |
| January 2018 sediment | 3.56 | N/A | -17 | N/A | N/A | N/A | N/A | N/A |

**S1 Table.** Physicochemical characteristics of water samples collected in July 28, 2017 and January 14, 2018 at EB-90M. Values with standard errors are an average of three distinct samples collected on the same day. N/A indicates that these parameters were not measured.
