# Supplementary material for: Characterization of an acid rock drainage microbiome and transcriptome at the Ely Copper Mine Superfund site
Source: PLoS One. 2020 Aug 12;15(8):e0237599. doi: 10.1371/journal.pone.0237599 (PMC7423320; doi:10.1371/journal.pone.0237599)
Supplement: S2 Table — Processing of sequence data with annotated taxonomy. No sequences were trimmed. The annotations made exclude unclassified or unassigned phyla, genera, species, and genes. (DOCX) [file pone.0237599.s003.docx]

|  | Total sequences | Total sequences annotated at phylum level | Total sequences annotated at genus level | Total sequences annotated at species level | Total sequences annotated for functional gene content |
| --- | --- | --- | --- | --- | --- |
| Jan_Sed1 (RNA) | 42120625 |  |  |  | 8431974 |
| Jan_Sed2 (RNA) | 43727285 |  |  |  | 8716922 |
| Jan_Sed3 (RNA) | 40783850 |  |  |  | 8582569 |
| July_Sed1 (RNA) | 40729825 |  |  |  | 8781516 |
| July_Sed2 (RNA) | 44323783 |  |  |  | 9338060 |
| July_Sed3 (RNA) | 42894787 |  |  |  | 9698186 |
| Jan_Sed1 (DNA) | 8657966 | 2273037 | 2041002 | 1868289 | 3950837 |
| Jan_Sed2 (DNA) | 13331856 | 3456212 | 3101554 | 2837243 | 6311126 |
| Jan_Sed3 (DNA) | 10472068 | 2717171 | 2440926 | 2233356 | 4970304 |
| July_Sed1 (DNA) | 18013801 | 5031440 | 4516036 | 4139272 | 10150791 |
| July_Sed2 (DNA) | 15854659 | 4273843 | 3834055 | 3520177 | 9081599 |
| July_Sed3 (DNA) | 17393085 | 4792847 | 4289974 | 3935174 | 10312495 |
| July_Water1 (DNA) | 15799031 | 3672688 | 3284370 | 3037485 | 7298852 |
| July_Water2 (DNA) | 25412320 | 6483330 | 5778525 | 5320405 | 14528972 |
| July_Water3 (DNA) | 13661993 | 2996148 | 2703716 | 2506361 | 5628858 |
| July_Water4 (DNA) | 17621356 | 3330132 | 3050608 | 2846327 | 7801989 |
| July_Water5 (DNA) | 17284817 | 3334637 | 3039405 | 2830526 | 7541842 |

**S2 Table.** Processing of sequence data with annotated taxonomy. No sequences were trimmed. The annotations made exclude unknown or unassigned phyla, genera, species, and genes.
