# Supplementary material for: Characterization of an acid rock drainage microbiome and transcriptome at the Ely Copper Mine Superfund site
Source: PLoS One. 2020 Aug 12;15(8):e0237599. doi: 10.1371/journal.pone.0237599 (PMC7423320; doi:10.1371/journal.pone.0237599)
Supplement: S3 Table — Shannon diversity indices assessing alpha diversity of bacterial taxa within July and January sediment and July water samples. (DOCX) [file pone.0237599.s004.docx]

| Summary | H-phylum | H-class | H-order | H-family | H-genus | H-species |
| --- | --- | --- | --- | --- | --- | --- |
| Jan Sed | 1.3 ± 0.003 | 2.2 ± 0.001 | 3.5 ± 0.002 | 4.3 ± 0.01 | 5.3 ± 0.002 | 6.8 ± 0.003 |
| July Sed | 1.3 ± 0.01 | 2.3 ± 0.02 | 3.6 ± 0.02 | 4.4 ± 0.03 | 5.4 ± 0.03 | 6.9 ± 0.02 |
| July Water | 1.2 ± 0.009 | 2.3 ± 0.08 | 3.6 ± 0.07 | 4.5 ± 0.06 | 5.5 ± 0.03 | 7.0 ± 0.08 |

**Table S3.** Shannon diversity indices assessing alpha diversity of bacterial taxa within July and January sediment and July water samples.
