# Supplementary material for: Characterization of an acid rock drainage microbiome and transcriptome at the Ely Copper Mine Superfund site
Source: PLoS One. 2020 Aug 12;15(8):e0237599. doi: 10.1371/journal.pone.0237599 (PMC7423320; doi:10.1371/journal.pone.0237599)
Supplement: S4 Table — Shannon diversity indices assessing alpha diversity of archaeal taxa within July and January sediment and July water samples. (DOCX) [file pone.0237599.s005.docx]

| Summary | H-Phylum | H-Class | H-Order | H-Family | H-Genus | H-Species |
| --- | --- | --- | --- | --- | --- | --- |
| Jan Sed | 0.59 ± 0.009 | 1.4 ± 0.004 | 2.4 ± 0.008 | 2.8 ± 0.01 | 3.9 ± 0.01 | 4.7 ± 0.02 |
| July Sed | 0.81 ± 0.02 | 1.5 ± 0.07 | 2.2 ± 0.1 | 2.5 ± 0.2 | 3.3 ± 0.3 | 4.0 ± 0.3 |
| July Water | 0.96 ± 0.1 | 1.9 ± 0.07 | 2.7 ± 0.02 | 3.1 ± 0.02 | 4.0 ± 0.07 | 4.9 ± 0.09 |

**Table S4.** Shannon diversity indices assessing alpha diversity of archaeal taxa within July and January sediment and July water samples.
