# Supplementary material for: Characterization of an acid rock drainage microbiome and transcriptome at the Ely Copper Mine Superfund site
Source: PLoS One. 2020 Aug 12;15(8):e0237599. doi: 10.1371/journal.pone.0237599 (PMC7423320; doi:10.1371/journal.pone.0237599)
Supplement: S5 Table — Shannon diversity indices assessing alpha diversity of fungal taxa within July and January sediment and July water samples. (DOCX) [file pone.0237599.s006.docx]

| Summary | H-phylum | H-class | H-order | H-family | H-genus | H-species |
| --- | --- | --- | --- | --- | --- | --- |
| Jan Sed | 0.63 ± 0.001 | 2.3 ± 0.008 | 3.3 ± 0.004 | 4.1 ± 0.005 | 4.8 ± 0.005 | 5.5 ± 0.009 |
| July Sed | 0.67 ± 0.003 | 2.3 ± 0.004 | 3.3 ± 0.001 | 4.0 ± 0.005 | 4.8 ± 0.009 | 5.4 ± 0.01 |
| July Water | 0.70 ± 0.01 | 2.3 ± 0.007 | 3.3 ± 0.004 | 4.1 ± 0.006 | 4.9 ± 0.005 | 5.5 ± 0.004 |

**Table S5.** Shannon diversity indices assessing alpha diversity of fungal taxa within July and January sediment and July water samples.
