# Supplementary material for: Characterization of an acid rock drainage microbiome and transcriptome at the Ely Copper Mine Superfund site
Source: PLoS One. 2020 Aug 12;15(8):e0237599. doi: 10.1371/journal.pone.0237599 (PMC7423320; doi:10.1371/journal.pone.0237599)
Supplement: S8 Table — The alpha diversity analysis of fungal taxa within sediment samples as well as beta diversity analyses across all sediment samples at different levels of annotation. The Shannon diversity index was determined to assess alpha diversity, and the ADONIS and ANOSIM analyses were used to determine the beta diversity among sediment from January 2018 compared to July 2017. Significance * ≤ 0.05, ** ≤ 0.01, *** ≤ 0.001. (DOCX) [file pone.0237599.s009.docx]

| Levels of Annotation | Alpha diversity:  Kruskal-Wallis  p value | Beta Diversity: adonis R^2^ | Beta Diversity:  adonis p value | Beta Diversity: ANOSIM R | Beta Diversity:  ANOSIM  p value |
| --- | --- | --- | --- | --- | --- |
| Phylum | 0.0495346* | 0.961 | 0.1 | 1 | 0.1 |
| Class | 0.827259 | 0.919 | 0.1 | 1 | 0.1 |
| Order | 0.0495346* | 0.867 | 0.1 | 1 | 0.1 |
| Family | 0.0495346* | 0.858 | 0.1 | 1 | 0.1 |
| Genus | 0.0495346* | 0.86 | 0.1 | 1 | 0.1 |

**S8 Table.** The alpha diversity analysis of fungal taxa within sediment samples as well as beta diversity analyses across all sediment samples at different levels of annotation. The Shannon diversity index was determined to assess alpha diversity, and the ADONIS and ANOSIM analyses were used to determine the beta diversity among sediment from January 2018 compared to July 2017. Significance * ≤ 0.05, ** ≤ 0.01, *** ≤ 0.001.
