# Supplementary material for: Characterization of an acid rock drainage microbiome and transcriptome at the Ely Copper Mine Superfund site
Source: PLoS One. 2020 Aug 12;15(8):e0237599. doi: 10.1371/journal.pone.0237599 (PMC7423320; doi:10.1371/journal.pone.0237599)
Supplement: S9 Table — Alpha diversity analysis of bacteria as well as the beta diversity analyses across all summer samples (i.e., water and sediment) at different levels of annotation. Significance * ≤ 0.05, ** ≤ 0.01, *** ≤ 0.001. (DOCX) [file pone.0237599.s010.docx]

| Levels of Annotation | Alpha diversity:  Kruskal-Wallis  p value | Beta Diversity: adonis R^2^ | Beta Diversity:  adonis  p value | Beta Diversity: ANOSIM R | Beta Diversity:  ANOSIM  p value |
| --- | --- | --- | --- | --- | --- |
| Phylum | 0.0526323 | 0.749 | 0.023* | 0.908 | 0.033* |
| Class | 0.654721 | 0.774 | 0.02* | 0.99 | 0.013* |
| Order | 0.456057 | 0.742 | 0.02* | 0.99 | 0.011* |
| Family | 0.456057 | 0.735 | 0.019* | 0.979 | 0.029* |
| Genus | 0.10105 | 0.724 | 0.014* | 0.959 | 0.016* |

**S9 Table.** Alpha diversity analysis of bacteria as well as the beta diversity analyses across all summer samples (i.e., water and sediment) at different levels of annotation. Significance * ≤ 0.05, ** ≤ 0.01, *** ≤ 0.001.
