# Supplementary material for: Characterization of an acid rock drainage microbiome and transcriptome at the Ely Copper Mine Superfund site
Source: PLoS One. 2020 Aug 12;15(8):e0237599. doi: 10.1371/journal.pone.0237599 (PMC7423320; doi:10.1371/journal.pone.0237599)
Supplement: S10 Table — Alpha diversity analysis of archaea as well as the beta diversity analyses across all summer samples (i.e., water and sediment) at different levels of annotation. Significance * ≤ 0.05, ** ≤ 0.01, *** ≤ 0.001. (DOCX) [file pone.0237599.s011.docx]

| Levels of Annotation | Alpha diversity:  Kruskal-Wallis  p value | Beta Diversity: adonis R^2^ | Beta Diversity:  adonis p value | Beta Diversity: ANOSIM R | Beta Diversity:  ANOSIM  p value |
| --- | --- | --- | --- | --- | --- |
| Phylum | 0.0253473* | 0.702 | 0.016* | 0.856 | 0.019* |
| Class | 0.0253473* | 0.743 | 0.018* | 1 | 0.019* |
| Order | 0.0253473* | 0.771 | 0.013* | 1 | 0.017* |
| Family | 0.0253473* | 0.760 | 0.012* | 1 | 0.017* |
| Genus | 0.0253473* | 0.770 | 0.022* | 1 | 0.023* |

**S10 Table.** Alpha diversity analysis of archaea as well as the beta diversity analyses across all summer samples (i.e., water and sediment) at different levels of annotation. Significance * ≤ 0.05, ** ≤ 0.01, *** ≤ 0.001.
