# Supplementary material for: Characterization of an acid rock drainage microbiome and transcriptome at the Ely Copper Mine Superfund site
Source: PLoS One. 2020 Aug 12;15(8):e0237599. doi: 10.1371/journal.pone.0237599 (PMC7423320; doi:10.1371/journal.pone.0237599)
Supplement: S12 Table — Summary of Prokka-annotated genes in water and sediment samples. All samples had 0 genes that were unassigned due to multi-mapping, duplication, nonjunctions, secondary structure, chimeras, fragment lengths, and mapping quality. (DOCX) [file pone.0237599.s013.docx]

|  | Assigned genes | Unassigned ambiguous genes | Unassigned genes without features | Unassigned unmapped genes |
| --- | --- | --- | --- | --- |
| Jan_Sed 1 (RNA) | 8431974 | 174613 | 6346153 | 6346153 |
| Jan_Sed 2 (RNA) | 8716922 | 210590 | 29703704 | 6500525 |
| Jan_Sed 3 (RNA) | 8582569 | 189702 | 25631418 | 7791056 |
| July_Sed1 (RNA) | 8781516 | 191619 | 27362315 | 5362556 |
| July_Sed2 (RNA) | 9338060 | 264165 | 29788694 | 5994227 |
| July_Sed3 (RNA) | 9698186 | 270397 | 28357387 | 5609499 |
| Jan_Sed 1 (DNA) | 3950837 | 651342 | 583259 | 3584348 |
| Jan_Sed 2 (DNA) | 6311126 | 889493 | 961323 | 5335088 |
| Jan_Sed 3 (DNA) | 4970304 | 712688 | 764463 | 4156851 |
| July_Sed1 (DNA) | 10150791 | 2361775 | 1329611 | 4536736 |
| July_Sed2 (DNA) | 9081599 | 1748193 | 1213910 | 4116664 |
| July_Sed3 (DNA) | 10312495 | 1853923 | 1326599 | 4207485 |
| July_Water1 (DNA) | 7298852 | 1848476 | 1406099 | 5511353 |
| July_Water2 (DNA) | 14528972 | 1452939 | 2508826 | 7354311 |
| July_Water3 (DNA) | 5628858 | 1517290 | 1124854 | 5616512 |
| July_Water4 (DNA) | 7801989 | 1502973 | 1683284 | 6944003 |
| July_Water5 (DNA) | 7541842 | 1538696 | 1575900 | 6907643 |

**S12 Table.** Summary of Prokka-annotated genes in water and sediment samples. All samples had 0 genes that were unassigned due to multi-mapping, duplication, nonjunctions, secondary structure, chimeras, fragment lengths, and mapping quality.
