# Supplementary material for: Characterization of an acid rock drainage microbiome and transcriptome at the Ely Copper Mine Superfund site
Source: PLoS One. 2020 Aug 12;15(8):e0237599. doi: 10.1371/journal.pone.0237599 (PMC7423320; doi:10.1371/journal.pone.0237599)
Supplement: S13 Table — Alignment statistics for raw reads (i.e., DNA and RNA) to metagenomic assembly in water and sediment samples. (DOCX) [file pone.0237599.s014.docx]

|  | Raw reads | Alignments of reads to assembly | Percent aligned | Sum of the counts of genes | Percent genes | Counts of gene expression | Entropy (base 2) |
| --- | --- | --- | --- | --- | --- | --- | --- |
| Jan_Sed 1 (RNA) | 42120625 | 35774472 | 0.849334 | 8431974 | 0.200186 | 725263 | 12.35 |
| Jan_Sed 2 (RNA) | 43727285 | 37226760 | 0.851339 | 8716922 | 0.199347 | 901553 | 13.865 |
| Jan_Sed 3 (RNA) | 40783850 | 32992794 | 0.808967 | 8582569 | 0.21044 | 884498 | 13.735 |
| July_Sed1 (RNA) | 40729825 | 35367269 | 0.868338 | 8781516 | 0.215604 | 650013 | 12.934 |
| July_Sed2 (RNA) | 44323783 | 38329556 | 0.864763 | 9338060 | 0.210678 | 885338 | 14.821 |
| July_Sed3 (RNA) | 42894787 | 37285288 | 0.869227 | 9698186 | 0.226092 | 818320 | 14.569 |
| Jan_Sed 1 (DNA) | 7459799 | 5073618 | 0.680128 | 3950837 | 0.529617 | 836723 | 18.791 |
| Jan_Sed 2 (DNA) | 10195891 | 7996768 | 0.784313 | 6311126 | 0.618987 | 1027402 | 18.988 |
| Jan_Sed 3 (DNA) | 9892509 | 6315217 | 0.638384 | 4970304 | 0.502431 | 952057 | 18.935 |
| July_Sed1 (DNA) | 9232681 | 13477065 | 1.45971 | 10150791 | 1.09944 | 1101167 | 19.115 |
| July_Sed2 (DNA) | 9075807 | 11737995 | 1.29333 | 9081599 | 1.00064 | 1108207 | 19.128 |
| July_Sed3 (DNA) | 9248528 | 13185600 | 1.4257 | 10312495 | 1.11504 | 1128098 | 19.092 |
| July_Water1 (DNA) | 10602665 | 10287678 | 0.970292 | 7298852 | 0.688398 | 1136184 | 18.68 |
| July_Water2 (DNA) | 11906406 | 18058009 | 1.51666 | 14528972 | 1.22027 | 1352115 | 18.882 |
| July_Water3 (DNA) | 11722195 | 8045481 | 0.686346 | 5628858 | 0.480188 | 1085013 | 18.931 |
| July_Water4 (DNA) | 11865343 | 10677353 | 0.899877 | 7801989 | 0.657544 | 1173064 | 18.761 |
| July_Water5 (DNA) | 10406350 | 10377174 | 0.997196 | 7541842 | 0.724735 | 1167074 | 18.822 |

**S13 Table.** Alignment statistics for raw reads (i.e., DNA and RNA) to metagenomic assembly in water and sediment samples.
