# Supplementary material for: Characterization of an acid rock drainage microbiome and transcriptome at the Ely Copper Mine Superfund site
Source: PLoS One. 2020 Aug 12;15(8):e0237599. doi: 10.1371/journal.pone.0237599 (PMC7423320; doi:10.1371/journal.pone.0237599)
Supplement: S14 Table — Taxa over- or underexpressing various crtB phytoene synthase genes with corresponding log fold change and interaction p-values (Type:Season). Any read that could not be taxonomically annotated does not have taxon listed. (DOCX) [file pone.0237599.s015.docx]

| Locus | Gene name | Contig | Genus | Winter RNA/Summer RNA  Log FC | P interaction |
| --- | --- | --- | --- | --- | --- |
| FHBHJPKI_508463 | *crtB_124* | NODE_39772 | *Azospirillum* | -1.08994422288445 | 0.0223 |
| FHBHJPKI_527473 | *crtB_132* | NODE_42893 | *Bradyrhizobium* | 2.69171903245963 | 0.823 |
| FHBHJPKI_552455 | *crtB_139* | NODE_47215 | *Dyella* | -1.15807911669374 | 0.340 |
| FHBHJPKI_589145 | *crtB_153* | NODE_53954 | *Sorangium* | 3.26078046614238 | 0.290 |
| FHBHJPKI_610054 | *crtB_159* | NODE_58096 | *Azospirillum* | -1.58128960356752 | 0.0123 |
| FHBHJPKI_751182 | *crtB_189* | NODE_90509 | *Sideroxydans* | 0.133258466704741 | 0.330 |
| FHBHJPKI_29949 | *crtB_19* | NODE_288 | *Candidatus* koribacter | 0.566939228604913 | 0.458 |
| FHBHJPKI_864289 | *crtB_207* | NODE_122814 |  | -0.702421586857663 | 0.616 |
| FHBHJPKI_878236 | *crtB_210* | NODE_127236 | *Saccharopolyspora* | -0.0482764991494405 | 0.906 |
| FHBHJPKI_1105971 | *crtB_241* | NODE_212938 |  | -0.111466841440426 | 0.879 |
| FHBHJPKI_1189965 | *crtB_252* | NODE_251646 |  | -2.0747014231823 | 0.179 |
| FHBHJPKI_1238658 | *crtB_258* | NODE_276063 |  | 0.107827771064879 | 0.377 |
| FHBHJPKI_58494 | *crtB_26* | NODE_797 | *Acidobacterium* | -1.48984654851084 | 0.449 |
| FHBHJPKI_1427001 | *crtB_282* | NODE_384361 |  | 5.62334655483126 | 1.54E-17 |
| FHBHJPKI_82616 | *crtB_33* | NODE_1382 | *Sphaerobacter* | -3.50962933632014 | 8.14E-06 |
| FHBHJPKI_103003 | *crtB_39* | NODE_1991 | *Streptomyces* (67% match) | 0.795475812644669 | 0.0424 |
| FHBHJPKI_225785 | *crtB_58* | NODE_8043 | *Caulobacter* | 5.21602158235578 | 0.0136 |
| FHBHJPKI_247344 | *crtB_67* | NODE_9546 | *Sulfuritalea* (50% match) | -1.19015161803801 | 0.891 |
| FHBHJPKI_248753 | *crtB_69* | NODE_9653 | *Burkholderia* (50% match) | 3.56873132394317 | 0.000134 |
| FHBHJPKI_250033 | *crtB_71* | NODE_9746 | *Bradyrhizobium* | 3.60510548898569 | 0.0280 |
| FHBHJPKI_19714 | *crtB_9* | NODE_154 | *Micromonospora* (67% match) | 4.32140524160459 | 5.58E-06 |
| FHBHJPKI_1015820 | *crtB_228* | NODE_175773 | *Komagataeibacter* | -4.74967283955641 | 1.21E-17 |

**S14 Table.** Taxa expressing various *crtB* phytoene synthase genes with corresponding log_2_ fold change (Log FC) and p-interaction values. Transcripts that could not be taxonomically annotated do not have taxa listed.
